# Inframe insertion and splice site variants in *MFGE8* associate with protection against coronary atherosclerosis

**DOI:** 10.1038/s42003-022-03552-0

**Published:** 2022-08-17

**Authors:** Sanni E. Ruotsalainen, Ida Surakka, Nina Mars, Juha Karjalainen, Mitja Kurki, Masahiro Kanai, Kristi Krebs, Sarah Graham, Pashupati P. Mishra, Binisha H. Mishra, Juha Sinisalo, Priit Palta, Terho Lehtimäki, Olli Raitakari, Estonian Biobank Research Team, Tõnu Esko, Andres Metspalu, Reedik Mägi, Mari Nelis, Lili Milani, The Biobank Japan Project, Koichi Matsuda, Yuji Yamanashi, Yoichi Furukawa, Takayuki Morisaki, Yoshinori Murakami, Yoichiro Kamatani, Kaori Muto, Akiko Nagai, Wataru Obara, Ken Yamaji, Kazuhisa Takahashi, Satoshi Asai, Yasuo Takahashi, Takao Suzuki, Nobuaki Sinozaki, Hiroki Yamaguchi, Shiro Minami, Shigeo Murayama, Kozo Yoshimori, Satoshi Nagayama, Daisuke Obata, Masahiko Higashiyama, Akihide Masumoto, Yukihiro Koretsune, Yukinori Okada, FinnGen, Aarno Palotie, Mark Daly, Bridget Riley-Gills, Howard Jacob, Dirk Paul, Heiko Runz, Sally John, Robert Plenge, Mark McCarthy, Julie Hunkapiller, Meg Ehm, Kirsi Auro, Caroline Fox, Anders Mälarstig, Katherine Klinger, Deepak Raipal, Tim Behrens, Robert Yang, Richard Siegel, Tomi Mäkelä, Jaakko Kaprio, Petri Virolainen, Antti Hakanen, Terhi Kilpi, Markus Perola, Jukka Partanen, Anne Pitkäranta, Juhani Junttila, Raisa Serpi, Tarja Laitinen, Johanna Mäkelä, Veli-Matti Kosma, Urho Kujala, Outi Tuovila, Raimo Pakkanen, Justin Wade Davis, Danjuma Quarless, Slavé Petrovski, Eleonor Wigmore, Adele Mitchell, Benjamin Sun, Ellen Tsai, Denis Baird, Paola Bronson, Ruoyu Tian, Yunfeng Huang, Joseph Maranville, Elmutaz Mohammed, Samir Wadhawan, Erika Kvikstad, Minal Caliskan, Diana Chang, Tushar Bhangale, Kirill Shkura, Victor Neduva, Xing Chen, Åsa Hedman, Karen S. King, Padhraig Gormley, Jimmy Liu, Clarence Wang, Ethan Xu, Franck Auge, Clement Chatelain, Deepak Rajpal, Dongyu Liu, Katherine Call, Tai-He Xia, Matt Brauer, Huilei Xu, Amy Cole, Jonathan Chung, Jaison Jacob, Katrina de Lange, Jonas Zierer, Mitja Kurki, Aki Havulinna, Juha Mehtonen, Priit Palta, Shabbeer Hassan, Pietro Della Briotta Parolo, Wei Zhou, Mutaamba Maasha, Susanna Lemmelä, Manuel Rivas, Arto Lehisto, Vincent Llorens, Mari E. Niemi, Henrike Heyne, Kimmo Palin, Javier Garcia-Tabuenca, Harri Siirtola, Tuomo Kiiskinen, Jiwoo Lee, Kristin Tsuo, Kati Kristiansson, Kati Hyvärinen, Jarmo Ritari, Miika Koskinen, Katri Pylkäs, Marita Kalaoja, Minna Karjalainen, Tuomo Mantere, Eeva Kangasniemi, Sami Heikkinen, Samuel Heron, Dhanaprakash Jambulingam, Venkat Subramaniam Rathinakannan, Nina Pitkänen, Lila Kallio, Sirpa Soini, Eero Punkka, Teijo Kuopio, Marco Hautalahti, Laura Mustaniemi, Mirkka Koivusalo, Sarah Smith, Tom Southerington, Aarno Palotie, Elisabeth Widen, Mark J. Daly, Samuli Ripatti

**Affiliations:** 1 Institute for Molecular Medicine Finland (FIMM), HiLIFE, University of Helsinki, Helsinki, Finland; 2 Department of Internal Medicine, University of Michigan, Ann Arbor, MI USA; 3 The Broad Institute of MIT and Harvard, Cambridge, MA USA; 4 Analytic and Translational Genetics Unit, Masfsachusetts General Hospital, Harvard Medical School, Boston, MA USA; 5 Department of Statistical Genetics, Osaka University Graduate School of Medicine, Suita, Japan; 6 Estonian Genome Centre, Institute of Genomics, University of Tartu, Tartu, Estonia; 7 Department of Clinical Chemistry, Faculty of Medicine and Health Technology, Tampere University, Tampere, Finland; 8 Finnish Cardiovascular Research Centre, Faculty of Medicine and Health Technology, Tampere University, Tampere, Finland; 9 Department of Clinical Chemistry, Fimlab Laboratories, Tampere, Finland; 10 Heart and Lung Center, Helsinki University Hospital and Helsinki University, Helsinki, Finland; 11 Research Centre of Applied and Preventive Cardiovascular Medicine, University of Turku, Turku, Finland; 12 Centre for Population Health Research, University of Turku, Turku University Hospital, Turku, Finland; 13 Department of Clinical Physiology and Nuclear Medicine, University of Turku, Turku, Finland; 14 Laboratory for Statistical Analysis, RIKEN Center for Integrative Medical Sciences, Yokohama, Japan; 15 Department of Public Health, Clinicum, Faculty of Medicine, University of Helsinki, Helsinki, Finland; 16 Laboratory of Genome Technology, Human Genome Center, Institute of Medical Science, The University of Tokyo, Tokyo, Japan; 17 Laboratory of Clinical Genome Sequencing, Graduate School of Frontier Sciences, The University of Tokyo, Tokyo, Japan; 18 Division of Genetics, The Institute of Medical Science, The University of Tokyo, Tokyo, Japan; 19 Division of Molecular Pathology, IMSUT Hospital Department of Internal Medicine, Institute of Medical Science, The University of Tokyo, Tokyo, Japan; 20 Department of Cancer Biology, Institute of Medical Science, The University of Tokyo, Tokyo, Japan; 21 Laboratory of Complex Trait Genomics, Graduate School of Frontier Sciences, The University of Tokyo, Tokyo, Japan; 22 Department of Public Policy, Institute of Medical Science, The University of Tokyo, Tokyo, Japan; 23 Department of Urology, Iwate Medical University, Iwate, Japan; 24 Department of Internal Medicine and Rheumatology, Juntendo University Graduate School of Medicine, Tokyo, Japan; 25 Department of Respiratory Medicine, Juntendo University Graduate School of Medicine, Tokyo, Japan; 26 Division of Pharmacology, Department of Biomedical Science, Nihon University School of Medicine, Tokyo, Japan; 27 Division of Genomic Epidemiology and Clinical Trials, Clinical Trials Research Center, Nihon University. School of Medicine, Tokyo, Japan; 28 Tokushukai Group, Tokyo, Japan; 29 Departmentof Hematology, Nippon Medical School, Tokyo, Japan; 30 Department of Bioregulation, Nippon Medical School, Kawasaki, Japan; 31 Tokyo Metropolitan Geriatric Hospital and Institute of Gerontology, Tokyo, Japan; 32 Fukujuji Hospital, Japan Anti-Tuberculosis Association, Tokyo, Japan; 33 The Cancer Institute Hospital of the Japanese Foundation for Cancer Research, Tokyo, Japan; 34 Center for Clinical Research and Advanced Medicine, Shiga University of Medical Science, Shiga, Japan; 35 Department of General Thoracic Surgery, Osaka International Cancer Institute, Osaka, Japan; 36 IIZUKA HOSPITAL, Fukuoka, Japan; 37 National Hospital Organization Osaka National Hospital, Osaka, Japan; 38 Abbvie, Chicago, IL USA; 39 Astra Zeneca, Cambridge, UK; 40 Biogen, Cambridge, MA USA; 41 Celgene, Summit, NJ, United States/Bristol Myers Squibb, New York, NY USA; 42 Genentech, San Francisco, CA USA; 43 GlaxoSmithKline, Brentford, UK; 44 Merck, Kenilworth, NJ USA; 45 Pfizer, New York, NY USA; 46 Sanofi, Paris, France; 47 Maze Therapeutics, San Francisco, CA USA; 48 Janssen Biotech, Beerse, Belgium; 49 Novartis, Basel, Switzerland; 50 HiLIFE, University of Helsinki, Finland, Finland; 51 Auria Biobank/University of Turku/Hospital District of Southwest Finland, Turku, Finland; 52 THL Biobank/The National Institute of Health and Welfare Helsinki, Helsinki, Finland; 53 Finnish Red Cross Blood Service/Finnish Hematology Registry and Clinical Biobank, Helsinki, Finland; 54 Helsinki Biobank/Helsinki University and Hospital District of Helsinki and Uusimaa, Helsinki, Finland; 55 Northern Finland Biobank Borealis/University of Oulu/Northern Ostrobothnia Hospital District, Oulu, Finland; 56 Finnish Clinical Biobank Tampere/University of Tampere/Pirkanmaa Hospital District, Tampere, Finland; 57 Biobank of Eastern Finland/University of Eastern Finland/Northern Savo Hospital District, Kuopio, Finland; 58 Central Finland Biobank/University of Jyväskylä/Central Finland Health Care District, Jyväskylä, Finland; 59 Business Finland, Helsinki, Finland; 60 Northern Savo Hospital District, Kuopio, Finland; 61 Northern Ostrobothnia Hospital District, Oulu, Finland; 62 Pirkanmaa Hospital District, Tampere, Finland; 63 Hospital District of Helsinki and Uusimaa, Helsinki, Finland; 64 Hospital District of Southwest Finland, Turku, Finland; 65 The National Institute of Health and Welfare Helsinki, Helsinki, Finland; 66 Central Finland Health Care District, Jyväskylä, Finland; 67 University of Jyväskylä, Jyväskylä, Finland; 68 University of Helsinki, Helsinki, Finland; 69 Finnish Red Cross Blood Service, Helsinki, Finland; 70 University of Stanford, Stanford, CA USA; 71 University of Tampere, Tampere, Finland; 72 University of Oulu, Oulu, Finland; 73 University of Eastern Finland, Kuopio, Finland; 74 University of Turku, Turku, Finland; 75 Finnish Biobank Cooperative—FINBB, Helsinki, Finland

**Keywords:** Cardiovascular genetics, Genome-wide association studies

## Abstract

Cardiovascular diseases are the leading cause of premature death and disability worldwide, with both genetic and environmental determinants. While genome-wide association studies have identified multiple genetic loci associated with cardiovascular diseases, exact genes driving these associations remain mostly uncovered. Due to Finland’s population history, many deleterious and high-impact variants are enriched in the Finnish population giving a possibility to find genetic associations for protein-truncating variants that likely tie the association to a gene and that would not be detected elsewhere. In a large Finnish biobank study FinnGen, we identified an association between an inframe insertion rs534125149 in *MFGE8* (encoding lactadherin) and protection against coronary atherosclerosis. This variant is highly enriched in Finland, and the protective association was replicated in meta-analysis of BioBank Japan and Estonian biobank. Additionally, we identified a protective association between splice acceptor variant rs201988637 in *MFGE8* and coronary atherosclerosis, independent of the rs534125149, with no significant risk-increasing associations. This variant was also associated with lower pulse pressure, pointing towards a function of *MFGE8* in arterial aging also in humans in addition to previous evidence in mice. In conclusion, our results suggest that inhibiting the production of lactadherin could lower the risk for coronary heart disease substantially.

## Abstract (web summary)

A genome-wide association study identifies *MFGE8* as protective against coronary atherosclerosis in European and East Asian populations.

## Introduction

Cardiovascular disease (CVD) is the leading cause of premature death and disability worldwide, with both genetic and environmental determinants^1,2^. The most common cardiovascular disease is coronary heart disease (CHD), including coronary atherosclerosis and myocardial infarction, among others. While genome-wide association studies (GWAS) have identified multiple genetic loci associated with cardiovascular diseases, exact genes driving these associations remain mostly uncovered^3^.

Owing to Finland’s population history, many deleterious and high-impact variants are enriched in the Finnish population giving a possibility to find genetic associations that would not be detected elsewhere^4^. Many studies have reported high-impact loss-of-function (LoF) variants associated with risk factors for CVD, such as blood lipid levels, thus impacting on the CVD risk remarkably. For example, high-impact LoF variants in genes *LPA*^4^*, PCSK9*^5^*, APOC3*^6^, and *ANGPTL4*^7^ have been shown to be associated with Lipoprotein(a), LDL-cholesterol (LDL-C), or triglyceride levels, and lowering the CVD risk.

Besides blood lipids, other risk factors for CVD include hypertension, smoking and the metabolic syndrome cluster components. The mechanism that links these risk factors to atherogenesis, however, remains incompletely elucidated. Many, if not all, of these risk factors, however, also participate in the activation of inflammatory pathways, and inflammation in turn can alter the function of artery wall cells in a manner that drives atherosclerosis^8^.

Using data from a sizeable Finnish biobank study FinnGen (*n* = 260,405), we identified an association with an inframe insertion rs534125149 in *MFGE8* and protection against coronary atherosclerosis and other representations of major coronary heart disease (CHD), such as myocardial infarction (MI). This variant is highly enriched in Finland, 70-fold compared to Non-Finnish Europeans (NFE) in the gnomAD genome reference database^9^ with AF of 3% in Finland. This association was also replicated in BioBank Japan (BBJ) and Estonian Biobank (EstBB). We also identified a splice acceptor variant rs201988637 in the same gene, which is also associated with protection against coronary atherosclerosis and other representations of major CHD, indicating that rs534125149 has very similar effect on CHD as a splice acceptor variant in *MFGE8*. Associations of both of these two variants in *MFGE8* were specific to CHD, and they did not significantly (*p* < 1.75 × 10^−5^) increase risk for any other disease, highlighting *MFGE8* as a potential drug target candidate.

## Results

### GWAS results for coronary atherosclerosis

**Fig. 1:**
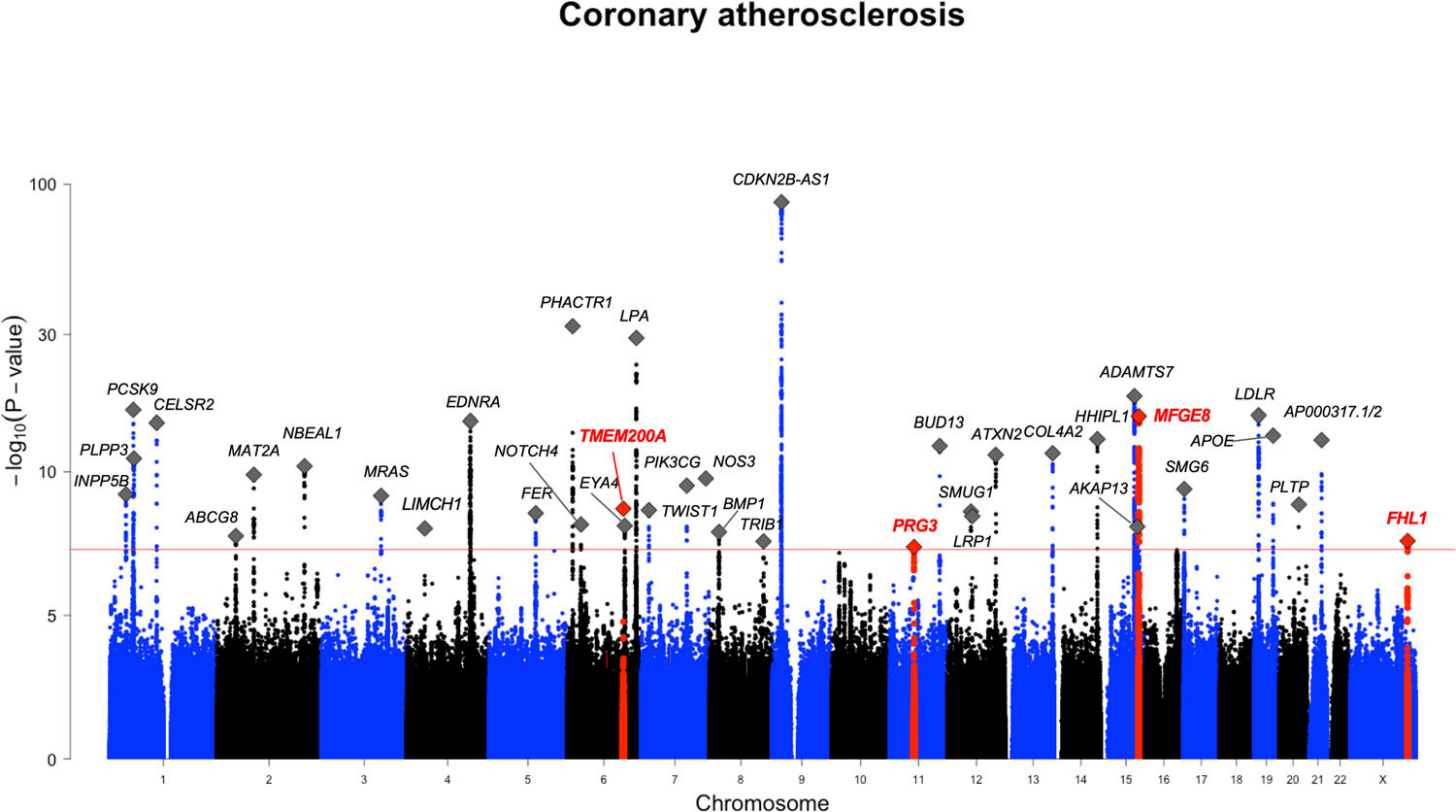
GWAS results for coronary atherosclerosis in FinnGen. Total number of independent genome-wide significant associations (GWS; *p* < 5 × 10^-8^) is 38, the lead variant in each marked with diamonds. Four previously unreported associations for CVD-related phenotypes are highlighted with ±750 Mb around the lead variant in the region as red and the lead variant marked with red diamond.

We identified a total of 2 302 variants associated (GWS, *p* < 5 × 10^−8^) with coronary atherosclerosis (detailed description of the definition of the endpoint is in Supplementary Note 1). These variants were located in 38 distinct genetic loci (a minimum of 0.5 Mb distance from each other; Fig. 1 and Supplementary Table 1). Out of the 38 GWS loci, four (within or near genes *MFGE8, TMEM200A, PRG3*, and *FHL1*) have not been previously reported to associate with any CVD-related endpoints or risk factor for CVD in GWAS Catalog^10^ [https://www.ebi.ac.uk/gwas/]. Lead variants in these loci and their characteristics are listed in Table 1 and locus zoom plots for each of the loci are in Supplementary Fig. 1.

**Table 1:** Lead variants in previously unreported loci for coronary atherosclerosis.

Lead variant chrom:pos:ref_alt (rsid)	Most severe consequence	Nearest gene	FIN enrichment (NFE)	AF	OR (*p*-value)	Info	# cs (Post-pr)	#coding in cs(s)
chr15:88901702:C_CTGT (rs534125149)	Inframe insertion	*MFGE8*	70.59	0.029	0.75 (2.60 × 10^−16^)	0.99	2 (0.705)	1
chr6:130483492:A_G (rs118042209)	Intergenic variant	*TMEM200A*	0.87	0.010	0.7 (1.90 × 10^−9^)	0.91	1 (0.904)	0
chr11:57380633:A:G (rs764568652)	Intron variant	*PRG3*	^a^	0.0003	7.72 (4.12 × 10^−8^)	0.89	1 (0.583)	0
chrX:136194941:C_G (rs5974585)	Intron variant	*FHL1*	1.25	0.49	0.95 (2.55 × 10^−8^)	0.99	1 (0.692)	0

^a^Variant not present in NFE in gnomAD.

Among these four previously unreported loci for coronary atherosclerosis, the locus near *MFGE8* had the strongest association (*p*-value = 2.63 × 10^−16^ for top variant rs534125149). The lead variant is an inframe insertion located in the sixth exon in the *MFGE8* gene (Supplementary Fig. 2) and it is highly enriched in the Finnish population compared to NFSEEs (Non-Finnish, Estonian or Swedish Europeans). Interestingly, *MFGE8* is mainly expressed in coronary and tibial arteries according to data from GTEx v8 (Supplementary Fig. 3), and furthermore the expression of *MFGE8* is highest in aorta. In addition, previously identified common variants in *MFGE8* locus that have been associated with decreased expression of *MFGE8* in tibial artery and aorta have also been associated with decreased risk of CHD^11^.

In addition to *MFGE8*, we identified three additional previously unreported loci to be associated with coronary atherosclerosis, *TMEM200A*, *PRG3* and *FHL1* being the nearest genes of the lead variants. *TMEM200A* and *PRG3* loci had one non-coding low-frequency variant reaching the genome-wide significance threshold, and *FHL1* had 11. All variants in the credible sets of all these associations were either intergenic or intronic variants and had no reported significant GWAS associations with any trait in the GWAS Catalog or significant eQTL associations in GTEx. The one variant (rs118042209) in the credible set of *TMEM200A* locus was associated with multiple disease endpoints representing major coronary heart disease (CHD) in FinnGen, including coronary atherosclerosis, ischemic heart disease and angina pectoris, whereas the lead variant in the *PRG3* locus was associated with cardiomyopathy. All variants in the credible set of *FHL1* were associated with multiple disease endpoints representing major CHD in FinnGen, including angina pectoris and ischemic heart disease. *TMEM200A* have been reported to be associated with ten traits (including height and trauma exposure) and *PRG3* with two traits (eosinophil count and eosinophil percentage of white cells) in the GWAS Catalog. *FHL1* gene had no reported associations in GWAS Catalog.

### Replication

**Fig. 2:**
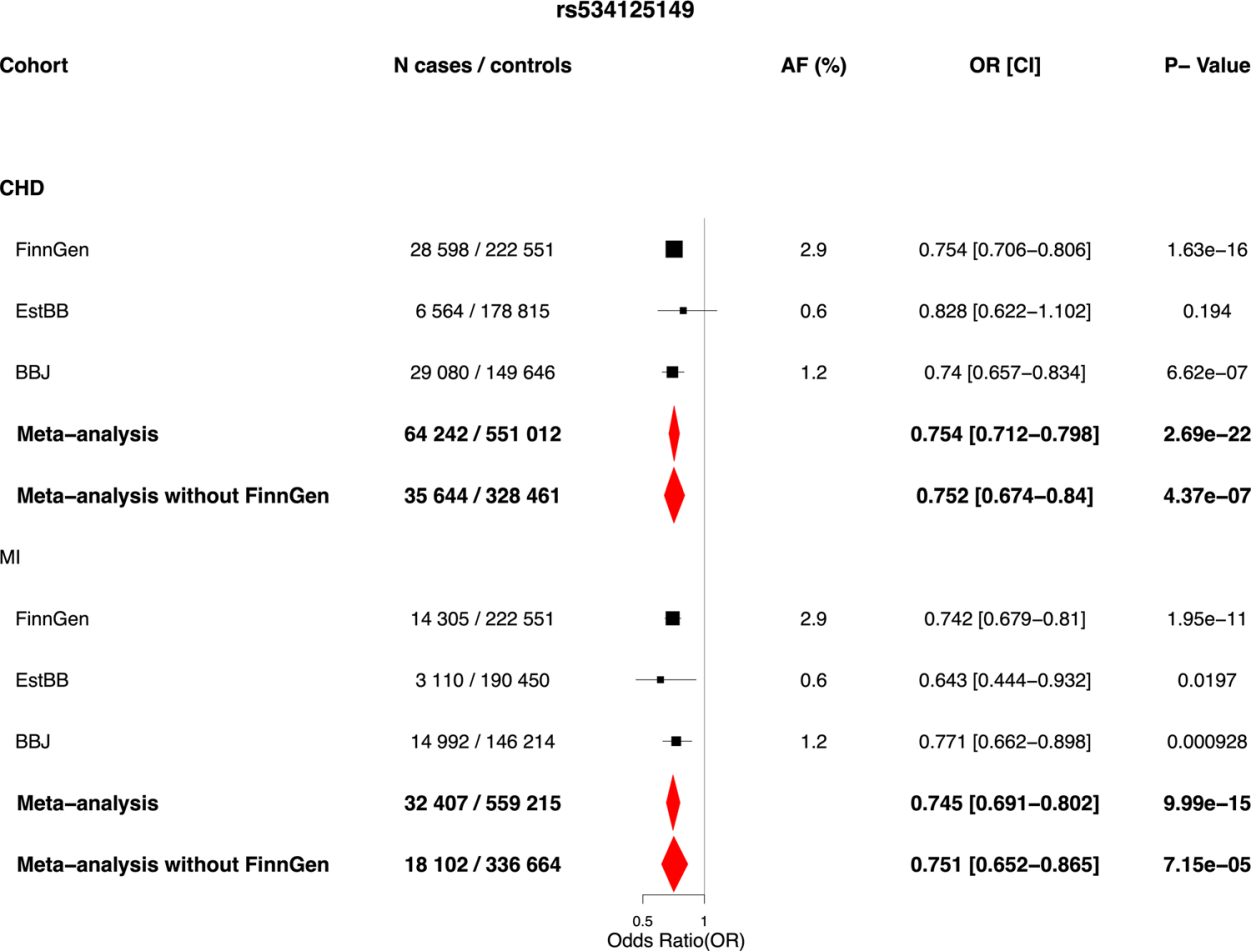
Results for rs534125149 against coronary heart disease and myocardial infarction across cohorts where available and meta-analysis results. Logistic regression has been applied, adjusted for age and sex. Meta-analysis was performed using inverse-variance weighted fixed-effects meta-analysis method. Black dots represents odds ratios, and lines 95% confidence interval from the the single cohorts and red diamonds represent the results from meta-analysis ends of the diamonds representing the ends of the 95% confidence interval. Source data for the figure is in Supplementary Data 1.

Association between rs534125149 in *MFGE8* locus with CHD was replicated in Biobank Japan^12,13^ (BBJ) and the Estonian Biobank (EstBB)^14^ (35,644 cases and 328 461 controls total: OR = 0.752 [0.67–0.84], *p* = 4.37 × 10^−7^). Association results for rs534125149 with CHD and MI across different cohorts are in Fig. 2. Post hoc power calculations for each cohort were performed (probability that the test will reject the null hypothesis H0 at GWS threshold) and the results as the function of effect size are in Supplementary Fig. 4. From these calculations we can see that in FinnGen the power to detect the variant as GWS is remarkably greater than in EstBB or BBJ, even with similar effect sizes and sample sizes. Therefore, this boost in power in FinnGen seems to be mainly due to a different allele frequencies, since this variant is highly enriched to Finland.

**Fig. 3:**
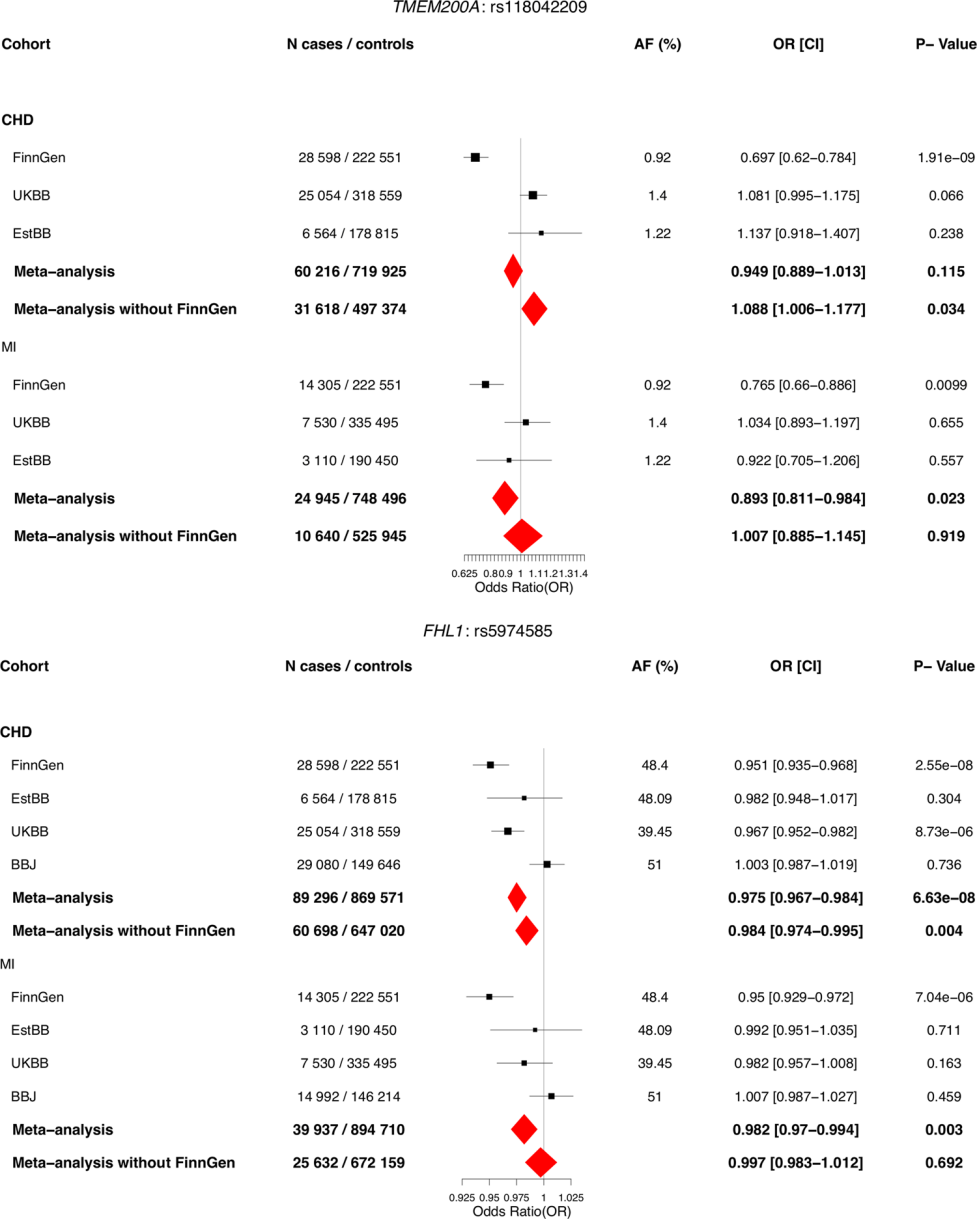
Results for rs118042209 in *TMEM200A* and rs5974585 in *FHL1* against coronary heart disease and myocardial infarction across different cohorts across cohorts where available. Logistic regression has been applied, adjusted for age and sex. Meta-analysis was performed using inverse-variance weighted fixed-effects meta-analysis method. Black dots represent odds ratios, and lines 95% confidence interval from the single cohorts and red diamonds represent the results from meta-analysis ends of the diamonds representing the ends of the 95% confidence interval. Source data for the figure is in Supplementary Data 1.

In addition to *MFGE8*, meta-analysis across FinnGen, UKBB, EstBB, and BBJ was performed for the lead variants in the three other previously unreported loci for CHD (*TMEM200A, PRG3*, and *FHL1*), where available. Lead variant in *PRG3* locus is highly enriched to Finland and absent in all other cohorts, and thus replication efforts for that variant were not possible. The two other loci that were meta-analyzed (*TMEM200A* and *FHL1*) did not replicate (*p*-value in the combined meta-analysis of the replication cohorts (meta-analysis without FinnGen) is smaller than 0.05/4 = 0.0125 and all effect size estimates are in same direction). Association results for rs534125149 with CHD and MI across different cohorts for *TMEM200A* and *FHL1* variants are in Fig. 3. Post hoc power calculations for each cohort were performed and the results as the function of effect size are in Supplementary Fig. 5. From those results we can see that the lack of replication in UKBB, EstBB and BBJ does not appear to be due to lack of power. Therefore, we identified and replicated one novel locus for CHD (*MFGE8)*.

### Phenome-wide association results for rs534125149

**Fig. 4:**
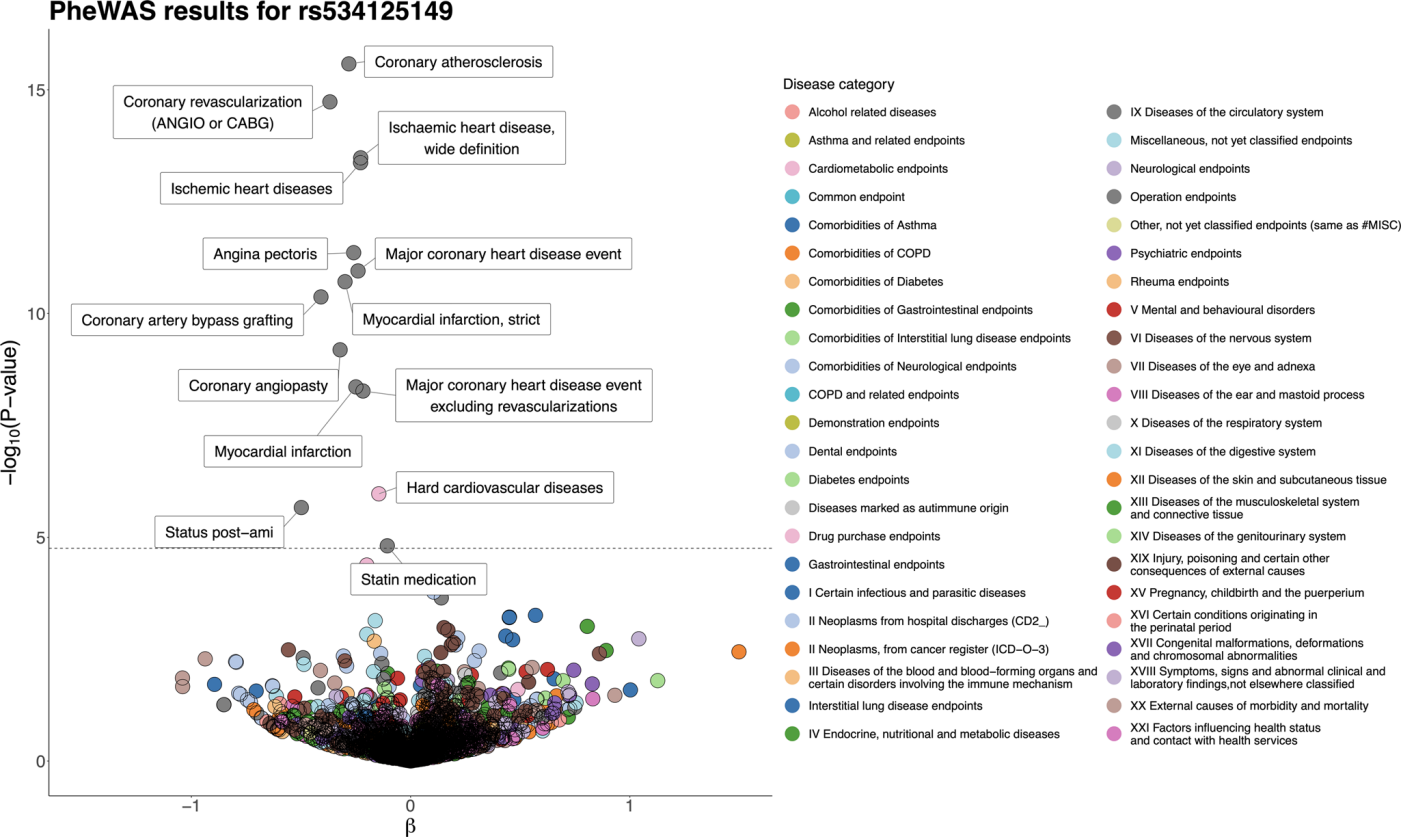
Phenome-wide association study (PheWAS) results for rs534125149. Total number of tested endpoints is 2861 (A complete list of endpoints analyzed and their definitions is available at https://www.finngen.fi/en/researchers/clinical-endpoints). The dashed line represents the phenome-wide significance threshold, multiple testing corrected by the number of endpoints = 0.05/2861 = 1.75 × 10^−5^. All endpoints reaching that threshold are labeled in the figure.

We observed a highly protective association for the Finnish enriched inframe insertion rs534125149 in the *MFGE8* gene and multiple disease endpoints, all representing major CHD, including coronary atherosclerosis (OR = 0.75 [0.71–0.81], *p* = 2.63 × 10^−16^) and myocardial infarction (MI) (OR = 0.74 [0.68–0.81], *p* = 1.95 × 10^−11^). In total, this variant was associated (PWS) with 14 disease endpoints, all representing major CHD (Fig. 4). Majority of them are highly overlapping, and thus similar associations to all of them is expected. Thus, we pruned the 14 PWS disease endpoints down to six disease endpoints (coronary atherosclerosis, coronary revascularization, ischemic heart diseases, major coronary heart disease event, myocardial infarction, and statin medication) that have fundamental characteristics for further analyses. For the inframe insertion rs534125149 in *MFGE8*, we did not detect other phenome-wide significant associations among the 2 861 endpoints in our data.

### Splice acceptor variant rs201988637 in *MFGE8*

**Fig. 5:**
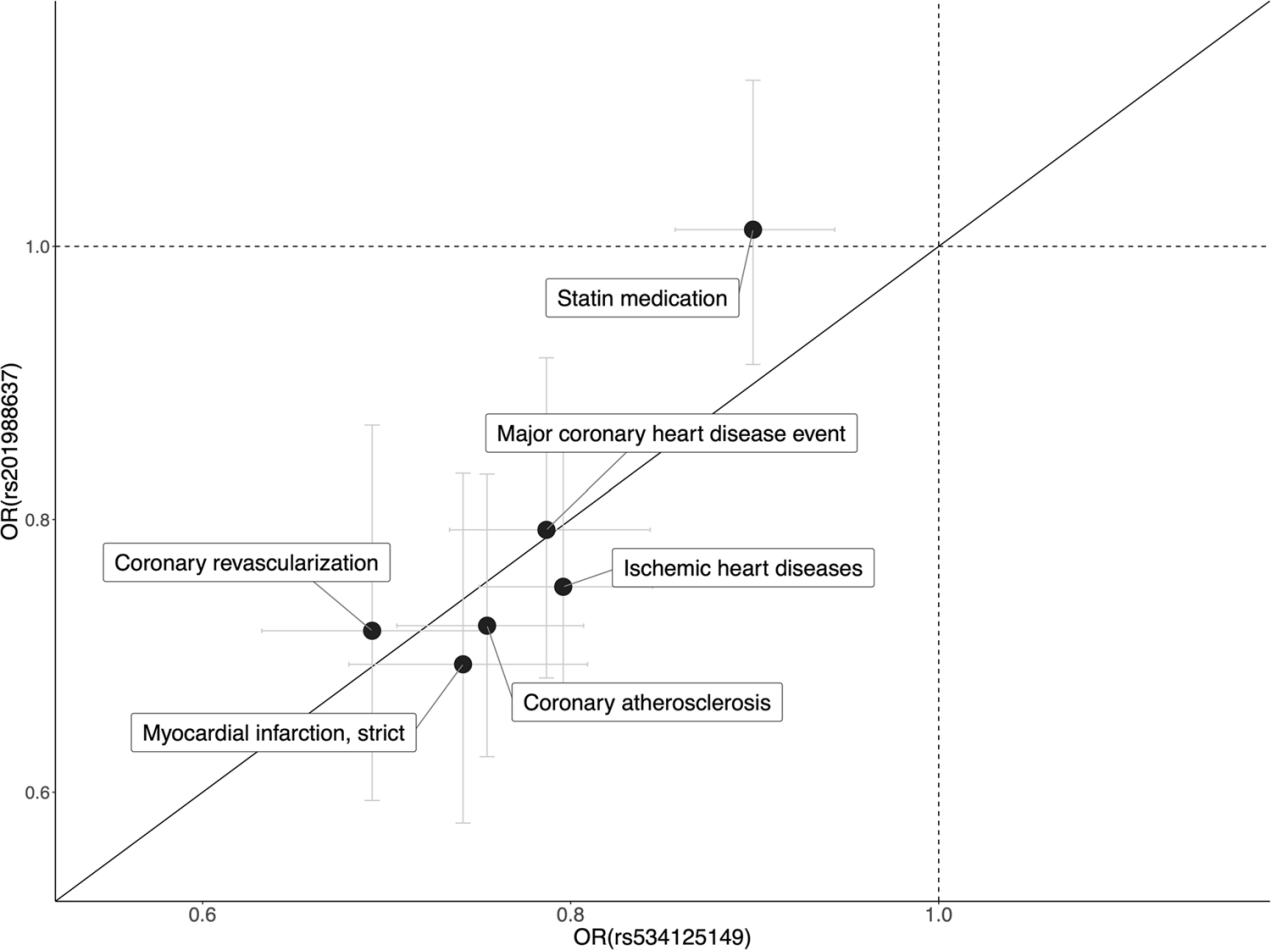
Effect size comparison. Comparison of the effects (OR) of rs534125149 and rs201988637 for 14 endpoints with *p*-value < 1.75 × 10^-5^ (PWS) for rs534125149 in FinnGen R6. 95% confidence intervals represented as gray lines.

In addition to inframe insertion rs53412514, we identified a splice acceptor variant (rs201988637) in MFGE8 to be associated with coronary atherosclerosis (OR = 0.72 [0.63–0.83], *p* = 7.94 × 10^−06^) and multiple disease endpoints representing major CHD. The splice acceptor variant had very similar PheWAS profile as the inframe insertion (Supplementary Fig. 6) and furthermore the two variants had very similar protective effect sizes for the endpoints (Fig. 5 and Supplementary Table 2). Similar to rs534125149, this variant is also highly enriched in Finland (37-fold compared to NFE), allele frequency in Finland being 0.6%. Moreover, both the splice acceptor and the inframe insertion variants were enriched to Eastern Finland (Supplementary Fig. 7).

These two variants (rs534125149 and rs201988637) are in low linkage disequilibrium (LD, *r*^2^ = 0.00015) and did not have any effect on the other variant’s associations with coronary atherosclerosis or MI (Table 2 and Supplementary Fig. 8). This indicates that they both are independently associated with these endpoints.

**Table 2:** Results of the conditional analysis on MI and coronary atherosclerosis.

Phenotype	SNPID [chr:position:ref:alt] (rsid)	Most severe consequence	Original GWAS results		Conditional results	
			OR [CI]	*p*-value	OR [CI]	*p*-value
Coronary atherosclerosis	chr15:88901702:C:CTGT (rs534125149)	Inframe insertion	0.75 [0.71–0.81]	2.63 × 10^−16^	0.75 [0.70–0.80]^a^	7.68 × 10^−15a^
	chr15:88899813:T:G (rs201988637)	Splice acceptor variant	0.72 [0.63–0.83]	7.94 × 10^−6^	0.73 [0.64–0.85]^b^	1.99 × 10^−5b^
Myocardial infarction, strict	chr15:88901702:C:CTGT (rs534125149)	Inframe insertion	0.74 [0.68–0.81]	1.95 × 10^−11^	0.79 [0.73–0.85]^a^	1.92 × 10^−10a^
	chr15:88899813:T:G (rs201988637)	Splice acceptor variant	0.69 [0.58–0.83]	9.62 × 10^−5^	0.71 [0.59–0.85]^b^	4.03 × 10^−4b^

This table present the conditional analysis results for coronary atherosclerosis and MI (strict definition, only primary diagnoses accepted) where the association has been conditioned on rs534125149 and rs201988637, separately.

^a^Conditional on rs201988637.

^b^Conditional on rs534125149.

### Survival analysis

**Fig. 6:**
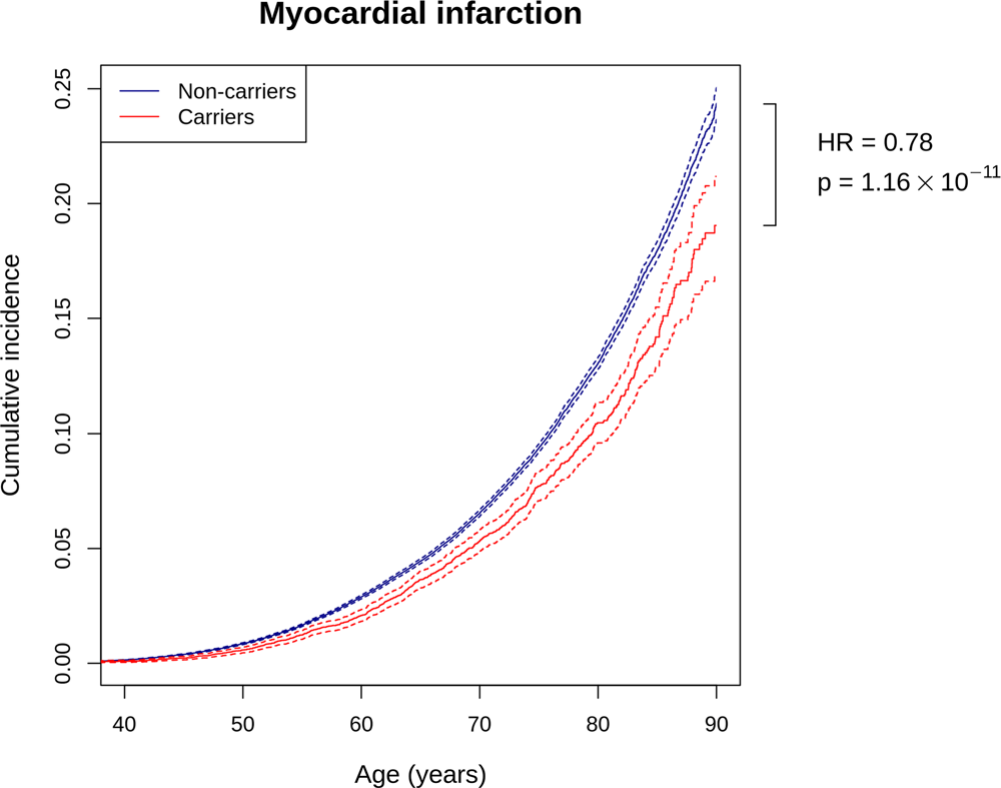
Cumulative incidence plots for first event of myocardial infarction in FinnGenR6. Red line represents carriers (homo- or heterozygous) for either rs534125149 or rs201988637 (*n* = 17,838), and blue line represent non-carriers (*n* = 242,567). Hazard ratio and *p*-value are from cox-proportional hazards model. Dashed lines represent 95% confidence intervals.

In addition to protection against coronary atherosclerosis and myocardial infarction, both the infame insertion rs534125149 and splice acceptor variant rs201988637 showed also significant association in survival analysis when analyzing survival time from birth to first diagnose of coronary atherosclerosis (HR = 0.78 [0.74–0.93]), *p* = 1.67 × 10^−17^ and HR = 0.77 [0.69–0.88], *p* = 5.08 × 10^−05^, respectively) and myocardial infarction (HR = 0.86 [0.80–0.93], *p* = 2.63 × 10^−10^ and HR = 0.72 [0.61–0.85], *p* = 8.16 × 10^−05^). In addition, when combining the heterozygous and homozygous carriers of both rs534125149 and rs201988637 together, carriers get the first diagnose significantly later than non-carriers (HR = 0.81 [0.77–0.85], *p* = 6.4 × 10^−16^ for coronary atherosclerosis and HR = 0.78 [0.72–0.85], *p* = 1.16 × 10^−11^ for MI) (Fig. 6).

In addition, as a sensitivity analysis we performed the similar Cox model for first event of MI by adding different risk factors for CHD as covariates in the model to see if any of these risk factors (BMI, Type 2 Diabetes, smoking, statin use or sex) have impact on the observed association. Risk factors were added to the model both individually and together. As a result, we saw only a small change in the effect size when adjusting for these risk factors (Supplementary Table 3). The change was more noticeable on *p*-values where the missing data in the added covariates lead to decreased statistical power.

### Associations with risk factors for CVD

**Fig. 7:**
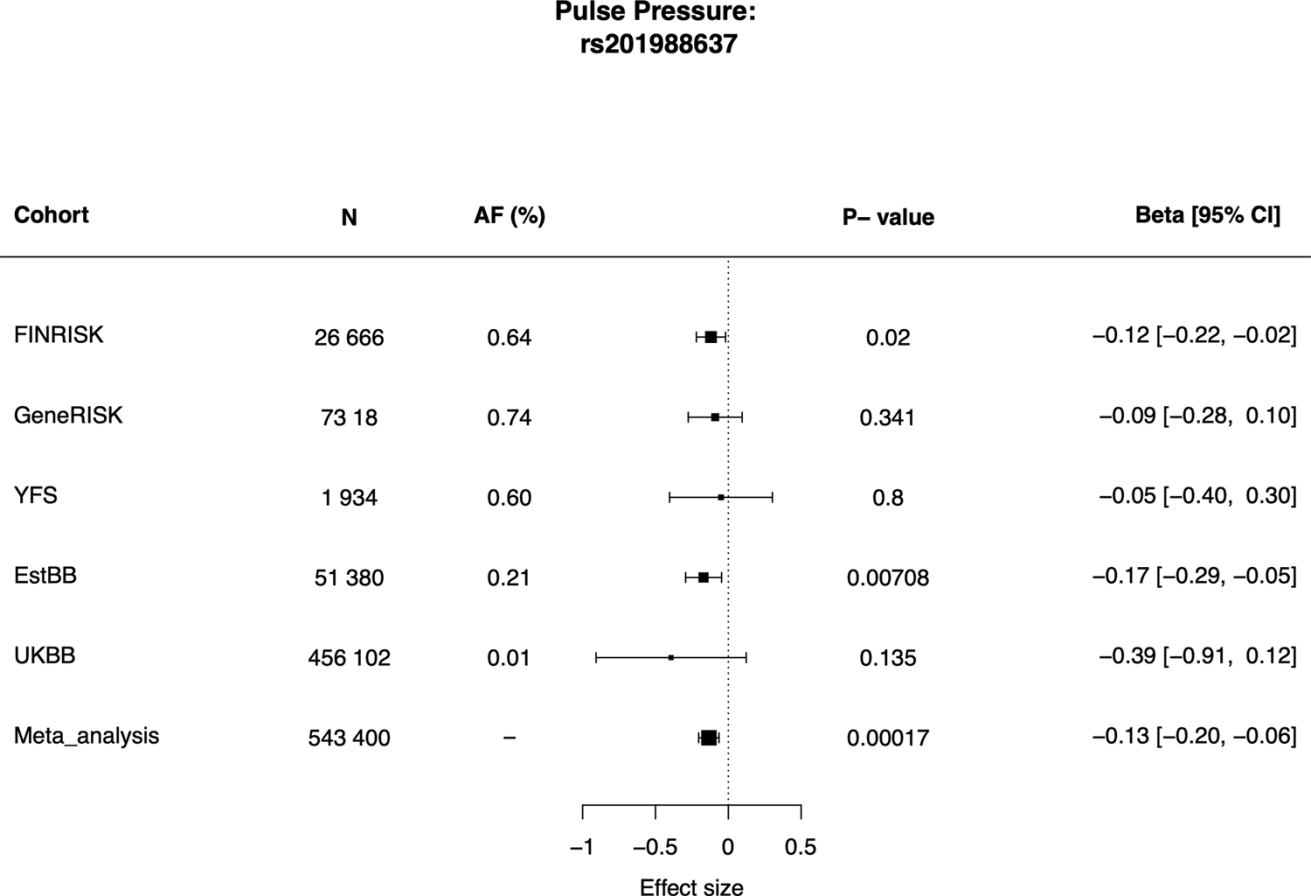
Results for pulse pressure association across all cohorts with splice acceptor variant rs201988637 available (FINRISK, GeneRISK, YFS, EstBB, and UKBB). Size of the boxes represent the sample size of the cohorts, and the lines the 95% confidence interval. Associations were tested using linear regression, adjusting for age and sex Pulse pressure phenotypes were inverse-rank normalized prior analysis. Source data for the figure is in Supplementary Data 1.

We then tested for possible associations between the *MFGE8* variants and risk factors for CVD. The splice acceptor variant rs201988637 was associated with pulse pressure in analyses across four cohorts with pulse pressure measurement and variant rs201988637 available, with the risk lowering allele associated with lower pulse pressure (*p* = 1.7 × 10^−04^, *β* = −0.13 [−0.2 to −0.06]) (Fig. 7). Association with pulse pressure was also tested for inframe insertion rs534125149 and previously reported common variant in the locus, rs8042271 across all where the variants were available. We saw consistent effect sizes across the cohorts, and significant (*p* < 0.05) meta-analysis *p*-values for both variants (Supplementary Fig. 9).

In addition, in recent studies for blood pressure measurements (systolic and diastolic blood pressure and pulse pressure), genome-wide significant association have been reported in the region^15,16^. To assess whether these reflects the same signal, we performed colocalization analysis in the region ±200 kB around rs53412514 using Coloc package in R^17^ with coronary atherosclerosis results from FinnGen and pulse pressure GWAS results from Evangelou et al.^16^ The probability for shared signal (PP4) was 97.1%, further validating *MFGE8* locus is associated with pulse pressure (Supplementary Fig. 10).

In addition to pulse pressure associations in the region, rs534125149 was significantly associated with height, but further analysis pointed this signal to be reflecting the association of a known association of *ACAN* with height, located near *MFGE8* (Supplementary Fig. 11). No associations with other risk factors were observed.

In the Corogene cohort (*n* = 4896), rs534125149 was significantly (*p* < 0.05) associated with lower risk for acute coronary syndrome and stable coronary heart disease (RR = 0.87 and 0.83, respectively) compared to healthy controls, but not with myocardial infarction without coronary artery occlusion (Supplementary Fig. 12). These results are in line with our findings regarding the specificity of the association of variants in *MFGE8* on atherosclerotic cardiovascular disorders. The *p*-value for the difference of the AFs of rs534125149 among patients with acute coronary syndrome or stable coronary heart disease and among MINOCA was, however, not significant (*p* = 0.78), which may due to lack of power. In addition, the cohort is very heterogeneous.

### Previously reported common variants near *MFGE8*

Previously, common intergenic variant (rs8042271) near *MFGE8* has been reported to associate with coronary heart disease (CHD) risk^3,18^. We replicate this association (OR = 0.90, *p* = 3.69 × 10^−10^ for coronary atherosclerosis) in FinnGen. LD between the common variant rs8042271 and the inframe insertion rs534125149 is 0.154. The LD characteristics for all three variants in *MFGE8* (rs534125149, rs201988637 and rs8042271) in FinnGen are in Supplementary Table 4. Common variant rs8042271 was in the 95% credible set for MI with the causal probability of 0.003 but was not included in the 95% credible sets for coronary atherosclerosis (Supplementary Tables 5 and  6). The conditional analyses of all three *MFGE8* variants showed that the association of the previously reported common variant rs8042271 can be explained by the inframe insertion variant rs534125149, but not vice versa, and that the association of the splice acceptor variant rs201988637 is independent of both rs534125149 and rs8042271. (Supplementary Table 7). This was the case also with previously reported common variant rs734780, showing very similar LD with rs534125149 (0.112) as rs8042271 (0.154).

### Fine-mapping of the *MFGE8* locus

In our fine-mapping analyses, MI had most probably one credible set (set of causal variants) of 32 variants with the highest posterior probability (posterior probability = 0.62), and coronary atherosclerosis had two credible sets of 6 and 45 variants, respectively, with the highest posterior probability (posterior probability = 0.74). For both MI and coronary atherosclerosis, rs534125149 had the highest probability of being causal (probability of being causal = 0.250 and 0.318, respectively) and was included in the first credible set (Supplementary Tables 5 and 6; and Supplementary Fig. 13). Splice acceptor variant rs201988637 was not included in the credible sets for either MI or coronary atherosclerosis, whereas previously reported common variant rs8042271 was included in the credible set for MI with the probability of being causal = 0.003 (Supplementary Table 6).

### Protein modeling

We predicted the impact of the insertion variant rs534125149 on the protein structure of *MFGE8* using AlphaFold^19^. The predicted conformational changes were localized to a loop region within the C2 domain, ~20 Å away from the key amino acids involved in membrane binding (Supplementary Fig. 14)^20,21^. This loop contains Asn238, which is known to be glycosylated^22^. It is possible that the insertion of an additional asparagine may lead to impaired glycosylation, which is important for protein folding, among other cellular processes^23^. The role of this region in the function of *MFGE8* hasn’t been previously described and it is therefore unclear how this variant would otherwise lead to an impact on *MFGE8* function. Thus, further experimental work is necessary to understand the mechanism by which this variant leads to protection against coronary atherosclerosis.

## Discussion

Here, we show that a Finnish enriched inframe insertion in *MFGE8* is associated with substantially lower risk of diseases representing major CHD, including myocardial infarction and coronary atherosclerosis. This variant was associated with CHD specifically, and no significant association was observed to other diseases in a phenome-wide search, even if this can be due to lower statistical power in rare disease endpoints. Splice acceptor variant rs201988637 in *MFGE8* was also associated with lower pulse pressure, but not with blood lipids, blood pressure or other known coronary heart disease risk factors.

Our findings allow us to draw several conclusions. First, *MFGE8* is a potential intervention target with specific effects on coronary heart disease. Specific protective association with the variants in *MFGE8* and CHD shows potential for efficacy of a treatment targeting MFGE8 protein or downstream products. Second, the lack of risk elevation in other diseases provide evidence on the potential safety of the intervention. Previously, the protective effect of loss-of-function variants have been reported for example for *PCSK9*^5^ and *APOC3*^6^, and in phase I, II and III trials, inhibition of PCSK9 have led to significantly decreased LDL-C levels, and in short-term trials, PCSK9 inhibitors have been well-tolerated and have had a low incidence of adverse effects^24^ Based on the phenome-wide association profile for the splice acceptor variant rs201988637, we hypothesize that inhibiting *MFGE8* could lower the CHD risk, if the variant can be proved to be loss-of-function in *MFGE8*.

An association of a splice acceptor variant rs201988637 in *MFGE8* with lower pulse pressure, a potential biomarker for arterial stiffness^25^, are very much in line with previous studies on MFGE8 and the inflammatory aging process of the arteries, highlighting the possible role of *MFGE8* in arterial aging and stiffness. The *MFGE8* gene encodes Milk-fat globule-EGF 8 (MFGE8), or lactadherin, which is an integrin-binding glycoprotein implicated in vascular smooth muscle cell (VSMC) proliferation and invasion, and the secretion of pro-inflammatory molecules^26,27^. Lactadherin is known to play important roles in several other biological processes, including apoptotic cell clearance and adaptive immunity^28^, which are known to contribute to the pathogenesis of ischemic stroke. Initially lactadherin was identified as a bridging molecule between apoptotic cells and phagocytic macrophages^29–31^, but growing evidence has indicated that it is a secreted inflammatory mediator that orchestrates diverse cellular interactions involved in the pathogenesis of various diseases, including vascular metabolic disorders and some tumors^32–36^ and cancers, such as breast^34,37^, bladder^38^, esophageal^39^ and colorectal cancer^40^. Recently, not only has MFG-E8 expression emerged as a molecular hallmark of adverse cardiovascular remodeling with age^41–44^, but MFG-E8 signaling has also been found to mediate the vascular outcomes of cellular and matrix responses to the hostile stresses associated with hypertension, diabetes, and atherosclerosis^45–49^.

Arterial inflammation and remodeling are linked to the pathogenesis of age-associated arterial diseases, such as atherosclerosis. Recently, lactadherin has been identified as a novel local biomarker for aging arterial walls by high-throughput proteomic screening, and it has been shown to also be an element of the arterial inflammatory signaling network^50^. The transcription, translation, and signaling levels of MFG-E8 are increased in aged, atherosclerotic, hypertensive, and diabetic arterial walls in vivo, as well as activated VSMCs and a subset of macrophages in vitro. During aging, both MFG-E8 transcription and translation increase within the arterial walls and hearts of various species, including rats, humans, and monkeys^44,51–53^, and MFG-E8 is markedly up-regulated in rat aortic walls with aging^44^. High levels of MFG-E8 have also been detected within endothelial cells, SMC, and macrophages of atherosclerotic aortae in both mice and humans^49,54^. Furthermore, in the advanced atherosclerotic plaques found in murine models, decreased macrophage MFG-E8 levels are associated with an inhibition of apoptotic cell engulfment, leading to the accumulation of cellular debris during the pathogenesis of atherosclerosis. Lactadherin has, however, in contrast shown tissue protection in various models of organ injury, including suppression of inflammation and apoptosis in intestinal ischemia in mice^55^, as well as inducing recovery from ischemia by facilitating angiogenesis^56^.

In addition, expression of *MFGE8* is highly enriched to tissues relevant to the reported association, such as aorta. Genes nearby *MFGE8*, including *ABHD2* and *HAPLN3*, are, however similarly to *MFGE8* enriched to arteries^18^. Therefore, they could play a role in atherosclerosis via coordinated gene network. In addition, recent studies have pointed toward the fact that lncRNA, called CARMAL, may regulate the expression of *MFGE8*^57^.

Our study does, however, have a few limitations. First, our primary association results come from Finnish population with considerable elevation in allele frequency in *MFGE8* variants among Finns. Therefore, the replication of the association in other populations has reduced statistical power. However, there were enough carriers combined in Japanese, Estonian and UK samples to replicate robustly both the protective association with coronary heart disease and for pulse pressure. Secondly, although our data shows association with pulse pressure, which has previously been linked to arterial stiffness, the direct effect of the genetic variants on arterial stiffness and arterial aging needs further evidence. Lastly, with our dataset, we have not been able to demonstrate that the two variants (rs534125149 and rs201988637) in *MFGE8* are loss-of-function variants, and thus further experimental work is required to validate our findings.

In conclusion, our results suggests that inhibiting production of lactadherin could reduce the risk for coronary atherosclerosis substantially and thus present *MFGE8* as a potential therapeutical target for atherosclerotic cardiovascular disease. Our study also highlights the potential of FinnGen, as a large-scale biobank study in isolated population to identify high-impact variants either very rare or absent in other populations.

## Methods

### Study cohort and data

We studied total of 2 861 disease endpoints in Finnish biobank study FinnGen (*n* = 260 405) (Table 3). FinnGen (https://www.finngen.fi/en) is a large biobank study that aims to genotype 500,000 Finns and combine this data with longitudinal registry data, including national hospital discharge, death, and medication reimbursement registries, using unique national personal identification numbers. FinnGen includes prospective epidemiological and disease-based cohorts as well as hospital biobank samples.

**Table 3:** Basic characteristics of the study cohort.

	All	Females	Males
*N* (%)	260,405	147,061 (56.47%)	113,344 (43.53%)
Age (mean (sd))	53.15 (17.55)	51.84 (17.71)	54.85 (17.19)
BMI (mean (sd))^a^	27.29 (5.36)	27.21 (5.83)	27.38 (4.76)
Statin use (*N* (%))	86,466 (33.2%)	40,422 (27.48%)	46,044 (40.62%)
Hypertension (*N* (%))	68,005 (26.11%)	33,420 (22.72%)	34,585 (30.51%)
Smoking (*N* (%))^b^	1733 (1.07%)	901 (0.96%)	832 (1.22%)
Coronary atherosclerosis	28,598 (11.38%)	9252 (6.87%)	19,346 (17.86%)
Myocardial infarction	14,305 (6.04%)	3958 (2.87%)	10,347 (10.42%)

^a^BMI is available only from 178,966 individuals.

^b^Smoking information is available only from 98,654 individuals.

### Definition of disease endpoints

All the 2861 disease-endpoint analyzed in FinnGen have been defined based on registry linkage to national hospital discharge, death, and medication reimbursement registries. Diagnoses are based on International Classification of Diseases (ICD) codes and have been harmonized over ICD codes 8, 9, and 10. More detailed lists of the ICD codes used for the disease-endpoints myocardial infarction and coronary atherosclerosis, which are discussed more in this study, are in Supplementary Note 1. A complete list of endpoints analyzed, and their definitions is available at https://www.finngen.fi/en/researchers/clinical-endpoints.

### Genotyping and imputation

FinnGen samples were genotyped with multiple Illumina and Affymetrix arrays (Thermo Fisher Scientific, Santa Clara, CA, USA). Genotype calls were made with GenCall and zCall algorithms for Illumina and AxiomGT1 algorithm for Affymetrix chip genotyping data batchwise. Genotyping data produced with previous chip platforms were lifted over to build version 38 (GRCh38/hg38) following the protocol described here: dx.doi.org/10.17504/protocols.io.nqtddwn. Samples with sex discrepancies, high-genotype missingness (>5%), excess heterozygosity (±4SD) and non-Finnish ancestry were removed. Variants with high missingness (>2%), deviation from Hardy–Weinberg equilibrium (*p* < 1 × 10^−6^) and low minor allele count (MAC < 3) were removed.

Pre-phasing of genotyped data was performed with Eagle 2.3.5 (https://data.broadinstitute.org/alkesgroup/Eagle/) with the default parameters, except the number of conditioning haplotypes was set to 20,000. Imputation of the genotypes was carried out by using the population-specific Sequencing Initiative Suomi (SISu) v3 imputation reference panel with Beagle 4.1 (version 08Jun17.d8b, https://faculty.washington.edu/browning/beagle/b4_1.html) as described in the following protocol: dx.doi.org/10.17504/protocols.io.nmndc5e. SISu v3 imputation reference panel was developed using the high-coverage (25–30x) whole-genome sequencing data generated at the Broad Institute of MIT and Harvard and at the McDonnell Genome Institute at Washington University, USA; and jointly processed at the Broad Institute. Variant callset was produced with Genomic Analysis Toolkit (GATK) HaplotypeCaller algorithm by following GATK best practices for variant calling. Genotype-, sample- and variant-wise quality control was applied in an iterative manner by using the Hail framework v0.2. The resulting high-quality WGS data for 3775 individuals were phased with Eagle 2.3.5 as described above. As a post-imputation quality control, variants with INFO score <0.7 were excluded.

### Association testing and replication

A total of 260,405 samples from FinnGen Data Freeze 6 with 2861 disease endpoints were analyzed using Scalable and Accurate Implementation of Generalized mixed model (SAIGE), which uses saddlepoint approximation (SPA) to calibrate unbalanced case-control ratios^58^. Models were adjusted for age, sex, genotyping batch and first ten principal components. All variants reaching genome-wide significance *p*-value threshold of 5 × 10^−8^ are considered as genome-wide significant (GWS), and all disease-endpoints reaching multiple testing corrected (for the number of endpoints tested = 2861) *p*-value threshold of 0.05/2861 = 1.75 × 10^−5^ were considered as phenome-wide significant (PWS).

Independent GWS loci for atherosclerosis were determined as adding ±0.5 Mb around each variant that reached the genome-wide significance threshold, overlapping regions were merged. The publicly available summary statistics from CARDIoGRAMplusC4D, a large meta-analysis of CHD involving 60,801 cases and 123,504 controls^3^ was used for assessing whether the locus has been previously reported to associate with CHD. In addition, NHGRI-EBI GWAS Catalog^10^ was used for assessing whether the locus has been previously reported to associate with any CVD-related endpoint or traditional risk factor for CVD, such as blood lipids, BMI and blood pressure. All loci that had not been reported to associate with CVD were fine-mapped using FINEMAP^59^ to determine the credible sets in each signal, and meta-analyzed across the cohorts (UKBB, EstBB and BBJ) where available to test their novelty.

In Corogene^60^ (*n* = 5300), a sub-cohort of FinnGen where participants have been collected as patients with coronary heart disease (CHD) and other related heart diseases, we tested the association of rs534125149 with sub-types of coronary heart disease: acute coronary syndrome, stable coronary heart disease (CHD) and MINOCA^61^ (myocardial infarction no coronary artery occlusion), by which we refer to patients that have had symptoms, ECG-changes and cardiac enzyme or troponine release suggesting acute coronary syndrome, but did not have coronary stenosis. The acute coronary syndrome was further divided into unstable Angina pectoris, non-ST segment elevation myocardial infarction (NSTEMI) and ST segment elevation myocardial infarction (STEMI). Associations were tested by calculating risk ratios (RR) for carriers vs. non-carriers of rs534125149 using non-CHD group always as controls and excluding the other tested groups from the analysis. *p*-values were calculated using *χ*^2^-test, and *p*-values < 0.05 were considered significant.

### Survival analysis

Survival analysis for coronary atherosclerosis and myocardial infarction was performed using GATE^62^, which accounts for both population structure and sample relatedness and controls type I error rates even for phenotypes with extremely heavy censoring. GATE transforms the likelihood of a multivariate Gaussian frailty model to a modified Poisson generalized linear mixed model (GLMM^63,64^), and to obtain well-calibrated *p*-values for heavily censored phenotypes, GATE uses the SPA to estimate the null distribution of the score statistic. For coronary atherosclerosis and myocardial infarction, survival time from birth to first diagnose was analyzed for both rs534125149 and rs201988637. Models were adjusted for age, sex, genotyping batch and first ten principal components, similarly to original GWAS analyses. In addition, cox-proportional hazards model was used for survival analysis for coronary atherosclerosis and myocardial infarction using a binary variable (carrier or non-carrier) for either inframe insertion rs534125149 or splice acceptor variant rs201988637.

### Biomarker analyses

We tested the association of the two *MFGE8* variants (rs534125149 and rs201988637) with quantitative measurements of cardiometabolic relevance or known risk factors for CVD in two sub-cohorts of FinnGen, the population-based national FINRISK study^65^ (*n* = 26,717) and GeneRISK^66^ (*n* = 7239). The associations were tested across 66 quantitative measurements of cardiometabolic relevance in FINRISK, and for 158 sub-lipid species in GeneRISK. In Young Finns Study (YFS)^67^ cohort (*n* = 1934), we tested the association of the two variants with three measurements of arterial relevance (carotid artery distensibility, pulse wave velocity, and pulse pressure).

In addition to Finnish cohorts described above, we tested the association of the two variants in Estonian Biobank data (EstBB)^14,68^, BioBank Japan (BBJ)^12,13^, and UK Biobank (UKBB)^69^. In EstBB (*n* = 51,388–137,722) we tested the associations of both variants with body mass index (BMI), systolic and diastolic blood pressure (SBP and DBP) and pulse pressure (PP), in BBJ in we tested the association of rs534125149 with 17 known quantitative risk factors for CVD and lastly, in the UKBB we tested the association of rs201988637 with 79 measurements of cardiometabolic relevance. In all of these biomarker analyses, a linear regression model adjusted for age and sex was used and for all quantitative risk factors rank-based inverse normal transformation was applied prior to analysis. Bonferroni corrected *p*-value threshold for the number of phenotypes tested was used to assess the significance of resulting associations in each cohort.

For biomarkers that showed significant association in any of the cohorts, we performed a meta-analysis across all cohorts the measurement was available. Meta-analysis was performed using inverse-variance weighted fixed-effects meta-analysis method^70,71^. Bonferroni corrected *p*-value for number of traits tested (*n* = 2) was used to assess the significance of resulting associations in meta-analysis.

### Height association

To assess whether the association of rs534125149 with height was due to the *MFGE8* gene, we first performed conditional analysis of height conditioning the association for rs534125149, the lead variant in FinnGen height GWAS (rs11630187) and for previously known height-associated variant in the locus, rs16942341^72^, separately. Conditioning the height association on rs534125149 did not have much effect on the association of the lead variant for height (rs11630187) in the region (*p*-value before conditioning = 5.07 × 10^−34^ and after conditioning = 1.19 × 10^−26^), whereas when conditioning on the lead variant for height (rs11630187) in the region, the smallest *p*-value in the region was 1.39 × 10^−15^ (for variant rs28564751). In addition, conditioning on either known height-associated variant rs16942341 or lead variant for height in FinnGen (rs11630187) did not affect on rs534125149’s association with height (*p*-value before conditioning = 8.04 × 10^−13^ and after conditioning = 3.14 × 10^−12^ and 2.75 × 10^−05^, respectively)

In addition, to assess whether the association of rs534125149 with atherosclerotic cardiovascular disease and height reflect the same signal, we performed colocalization analysis in the region ±200kB around rs53412514 using Coloc package in R. The probability for shared signal (PP4) was 9.22 × 10^−13^, whereas probability for two independent (PP3) signals was 1, indicating two independent signals for height and coronary atherosclerosis in the locus.

### Identifying causal variants

We used FINEMAP^59^ on the GWAS summary statistics to identify causal variants underlying the associations for MI (strict definition, i.e., only primary diagnoses accepted) and coronary atherosclerosis. FINEMAP analyses were restricted to a ±1.5 Mb region around the rs534125149. We assessed variants in the top 95% credible sets, i.e., the sets of variants encompassing at least 95% of the probability of being causal (causal probability) within each causal signal in the genomic region. Credible sets were filtered if minimum linkage disequilibrium (LD, *r*^2^) between the variants in the credible set was <0.1, i.e., not clearly representing one signal.

### Protein modeling

The predicted structure of lactadherin was obtained from AlphaFold^19^ (https://alphafold.ebi.ac.uk/entry/Q08431). Model confidence for the domain containing the variant of interest was scored mostly as very high and was structurally similar to the crystal structure of bovine lactadherin^21^ (PDB ID:2PQS). The structure of the insertion variant rs534125149 was predicted using the AlphaFold Colab notebook (https://colab.research.google.com/github/deepmind/alphafold/blob/main/notebooks/AlphaFold.ipynb). Protein structures were visualized using PyMOL^73^.

### Reporting summary

Further information on research design is available in the Nature Research Reporting Summary linked to this article.

## Supplementary information

Supplementary Information

Supplementary Data 1

Reporting Summary

## Data Availability

Full GWAS results are publicly available through FinnGen PheWEB browser (r6.finngen.fi) and also at Open Targets website. The Finnish biobank data can be accessed through the Fingenious® services (web link: https://site.fingenious.fi/en/, email: contact@finbb.fi) managed by FINBB. The UK Biobank resource is available to bona fide researchers for health-related research in the public interest at https://www.ukbiobank.ac.uk/researchers/. The BBJ summary statistics are available at the National Bioscience Database Center (NBDC) Human Database (accession code: hum0197) and at the GWAS catalog (https://www.ebi.ac.uk/gwas/home). They are also browseable at our PheWeb website (https://pheweb.jp/). The variant rs534125149 was originally excluded from the publicly available GWAS summary statistics. Its associations were reported in Supplementary Fig. 4. The BBJ genotype data is accessible on request at the Japanese Genotype–phenotype Archive (http://trace.ddbj.nig.ac.jp/jga/index_e.html) with accession code JGAD00000000123 and JGAS00000000114. Genotype and phenotype data from the Estonian Biobank are available (https://genomics.ut.ee/en/biobank.ee/data-access) upon request. The dataset supporting the conclusions of this article were obtained from the Cardiovascular Risk in Young Finns Study, which comprises health-related participant data. The use of data is restricted under the regulations on professional secrecy (Act on the Openness of Government Activities, 612/1999) and on sensitive personal data (Personal Data Act, 523/1999, implementing the EU data protection directive 95/46/EC). Owing to these restrictions, the data cannot be stored in public repositories or otherwise made publicly available. Data access may be permitted on a case-by-case basis upon request only. Data sharing outside the group is done in collaboration with YFS group and requires a data-sharing agreement. Investigators can submit an expression of interest to the chairman of the publication committee Professor Mika Kähönen (Tampere University, Finland) or Professor Terho Lehtimäki (Tampere University, Finland).

## Code availability

The full genotyping and imputation protocol for FinnGen is described at dx.doi.org/10.17504/protocols.io.nmndc5e. The code used for the analyses in this paper are available from the corresponding author upon reasonable request.
