# Supplementary material for: Inframe insertion and splice site variants in MFGE8 associate with protection against coronary atherosclerosis
Source: Commun Biol. 2022 Aug 17;5:802. doi: 10.1038/s42003-022-03552-0 (PMC9385630; doi:10.1038/s42003-022-03552-0)
Supplement: Supplementary file 1 — Supplementary Information [file 42003_2022_3552_MOESM1_ESM.pdf]

Supplementary material

Supplementary Note 1: Definition of the disease endpoints

Detailed definition for the endpoints that are discussed in this study: coronary atherosclerosis, myocardial infarction (only main diagnoses accepted), ischemic heart diseases, coronary revascularization, statin medication and major coronary heart disease event.

Coronary atherosclerosis: Coronary atherosclerosis was defined as underlying or direct cause of death or as the main or side diagnosis at hospital discharge with ICD codes I24, I25, Z951 and T822 (ICD-10) or 414 and 9960A (ICD-9) or 414 (ICD-8). Samples with ICD code I253 (ICD-10) as underlying or direct cause of death or as the main or side diagnosis at hospital discharge were excluded from the cases. Samples with ischemic heart diseases (defined as underlying or direct cause of death or as the main diagnosis at hospital discharge with ICD codes I20-25 in ICD10) were removed from controls.

Myocardial infarction (strict definition): Myocardial infarction was defined as underlying or direct cause of death or as the main diagnosis at hospital discharge with ICD codes I21 and I22 (ICD-10) or 410 (ICD-9 and 8). Samples with ICD codes I2190, I2197, I2290 or I2297 (ICD-10) as underlying or direct cause of death or as the main diagnosis at hospital discharge were excluded from the cases.

Samples with ischemic heart diseases (defined as underlying or direct cause of death or as the main diagnosis at hospital discharge with ICD codes I20-25 in ICD10) were removed from controls.

Ischemic heart diseases: Ischemic heart diseases was defined as the main or side diagnosis at hospital discharge with ICD codes I20-25 (ICD-10), 410-414 (ICD-9 and 8) or as underlying or direct cause of death with ICD codes I21-25, I46, R96 or R98 (ICD-10), 410-414 or 798 (ICD-9 and 8). Samples with underlying or direct cause of death with ICD code 7980A were removed from cases.

Coronary revascularization: Coronary revascularization was defined as either coronary angioplasty or coronary artery bypass grafting with Samples with ischemic heart diseases (defined as underlying or direct cause of death or as the main diagnosis at hospital discharge with ICD codes I20-25 in ICD-10) were removed from controls.

Statin medication: Statin medication was defined as at least three prescription medicine purchases with ATC class C10AA.

Major coronary heart disease event: Major coronary heart disease event was defined as the main diagnosis at hospital discharge with ICD codes I200, I21 and I22 (ICD-10) or 410 and 411 (ICD-9 and 8) or direct cause of death with ICD codes I21-I25, I46m R96 or R98 (ICD-10) or 410-414 or 798 (ICD-9 and 8). Samples with direct cause of death with ICD 7980A (ICD9) were excluded from cases.

Supplementary figures

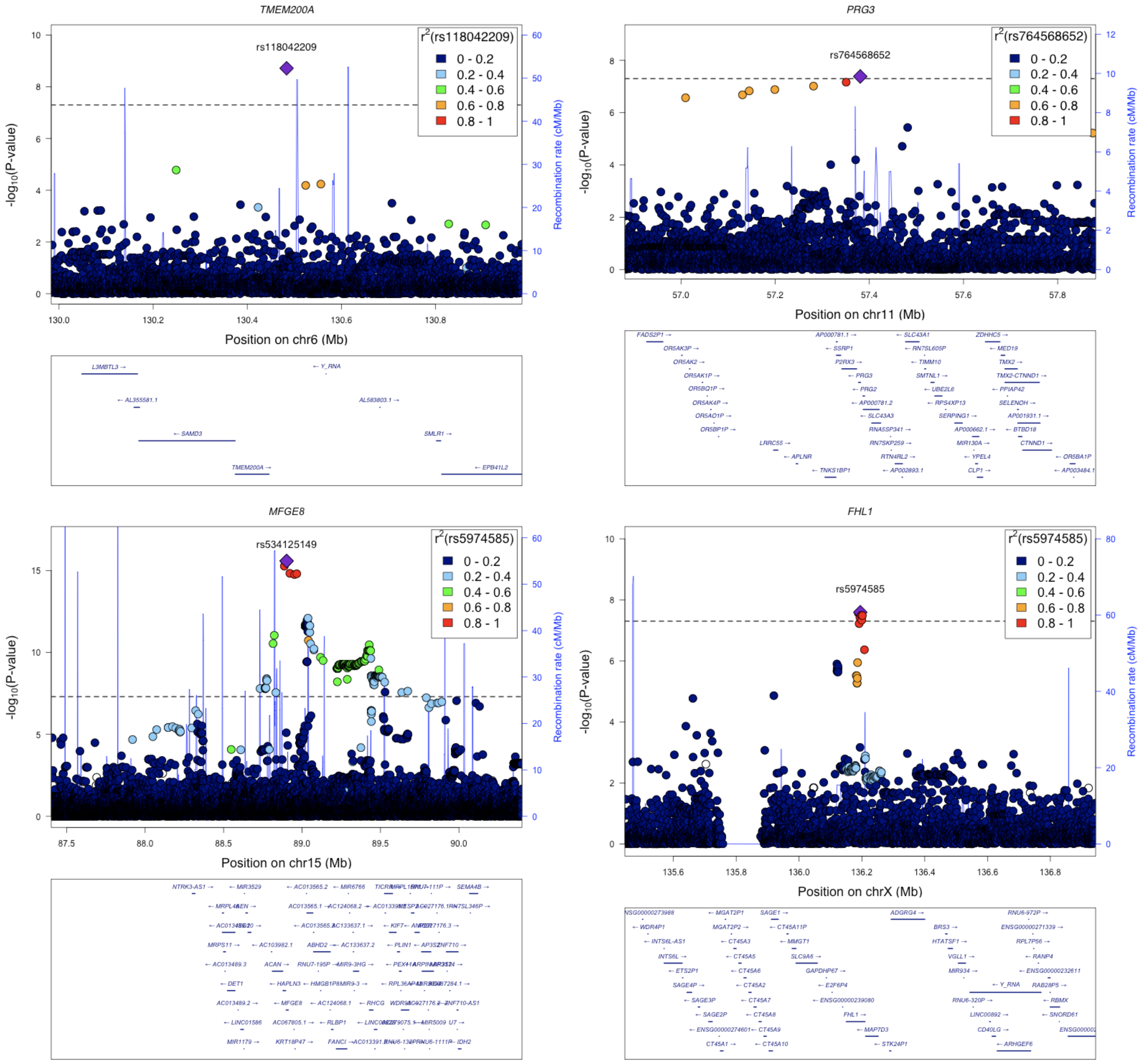

Supplementary Figure 1: *Locuszoomplots for the 4 previously unreported loci for CVD-related endpoints.*

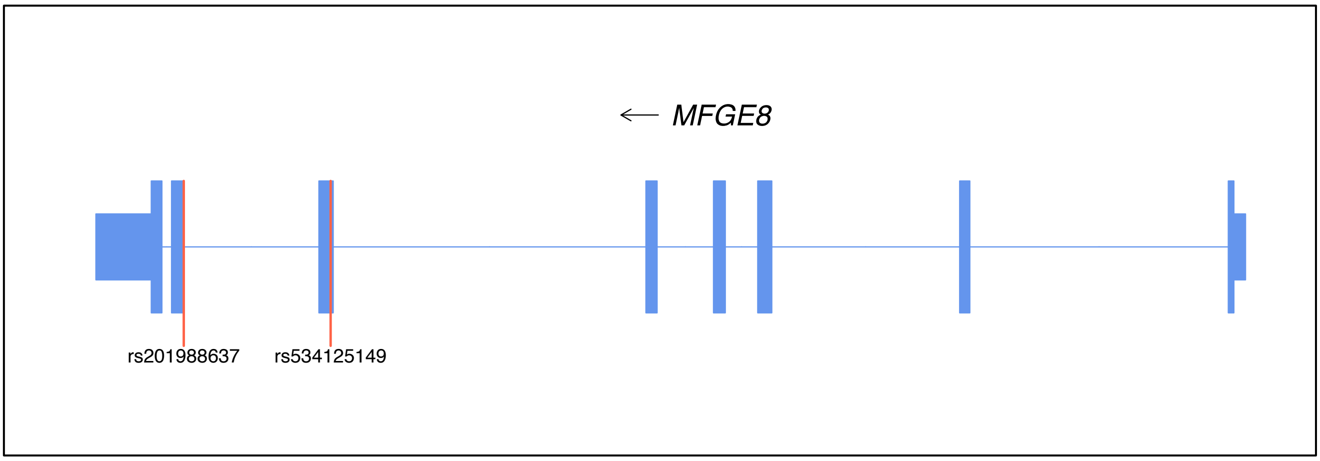

Supplementary Figure 2: *MFGE8 gene structure, inframe insertion (rs534125149) and splice acceptor variant (*rs201988637*) highlighted. Data extracted from UCSC browser (https://genome.ucsc.edu).*

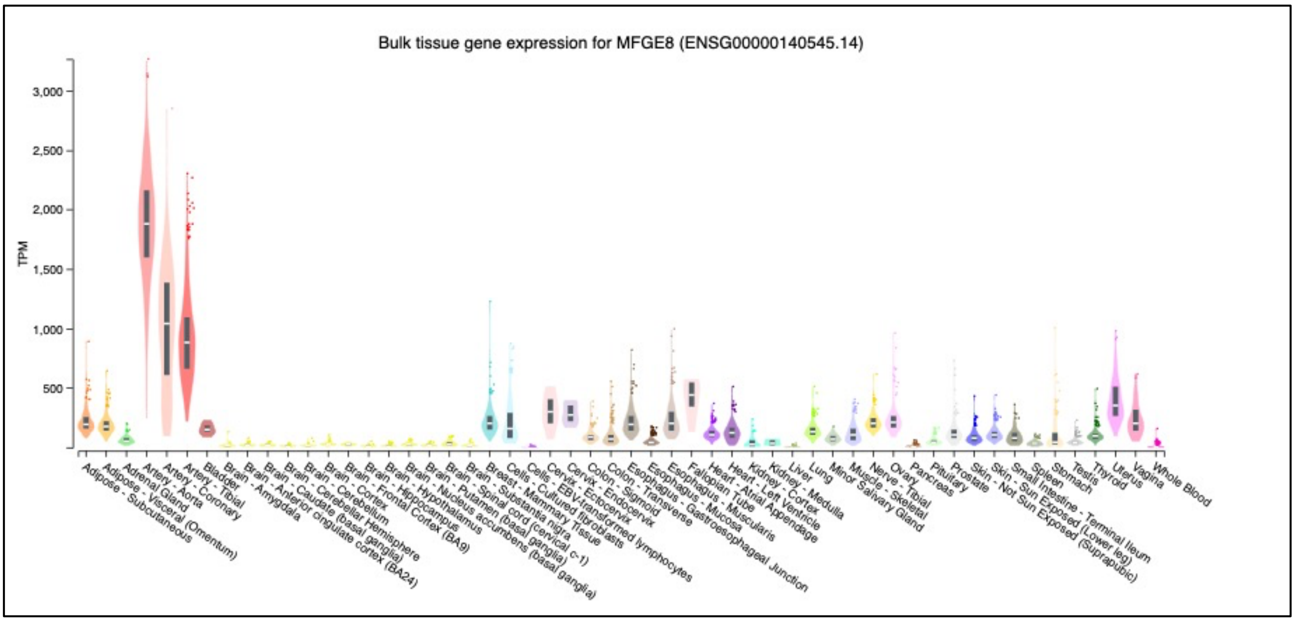

Supplementary Figure 3: *Gene expression for MFGE8 gene (Figure from GTEX v8 browser: https://gtexportal.org/home/gene/MFGE8). Boxplots are shown as median and 25th and 75th percentiles, and points are displayed as outliers if they are above or below 1.5 times the interquartile range.*

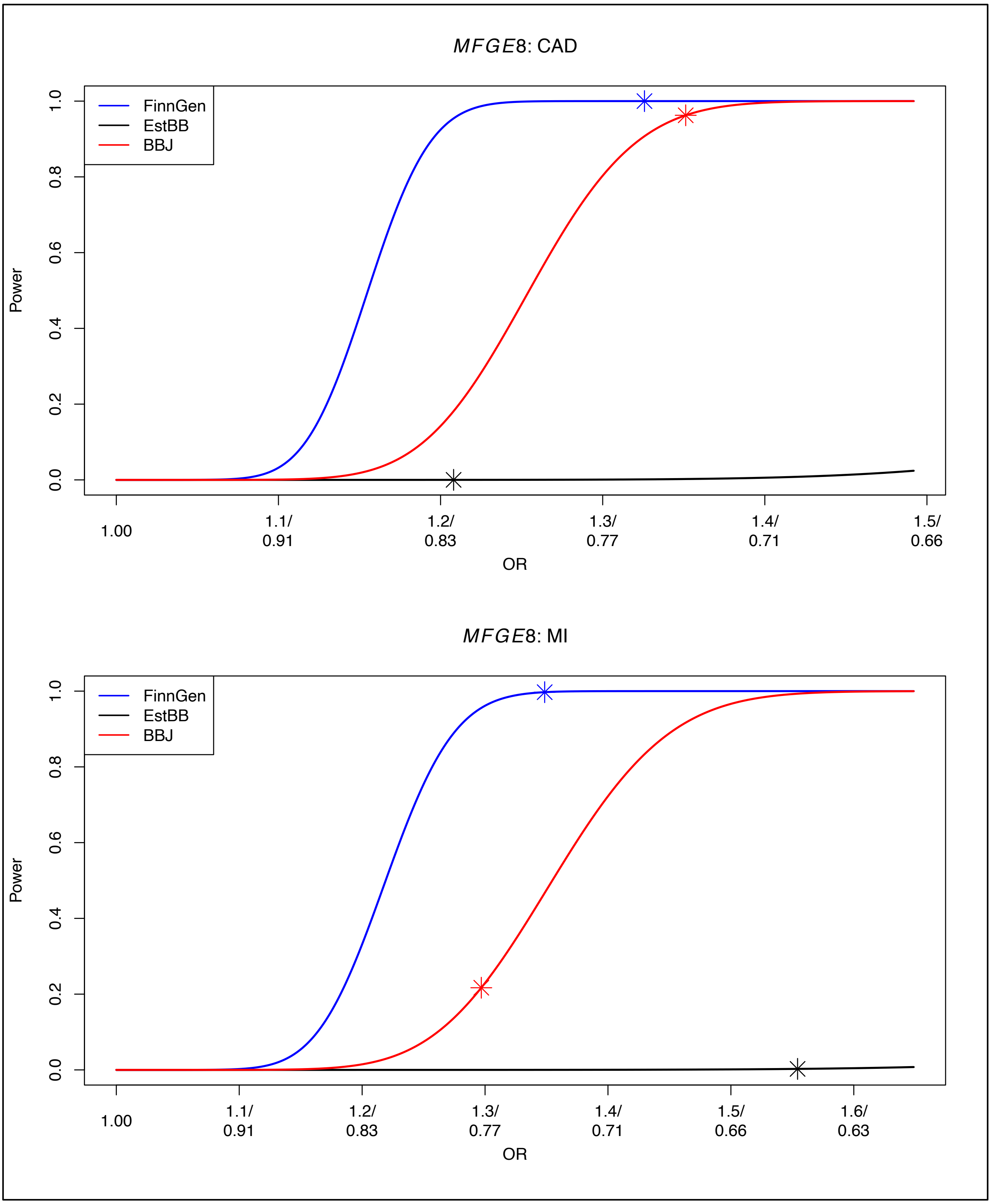

Supplementary Figure 4 (legend): *Post hoc power calculations for rs534125149. Power was calculated as a function of effect size for each cohort scenario (N cases and controls, AF). The actual effect sizes are marked as stars.*

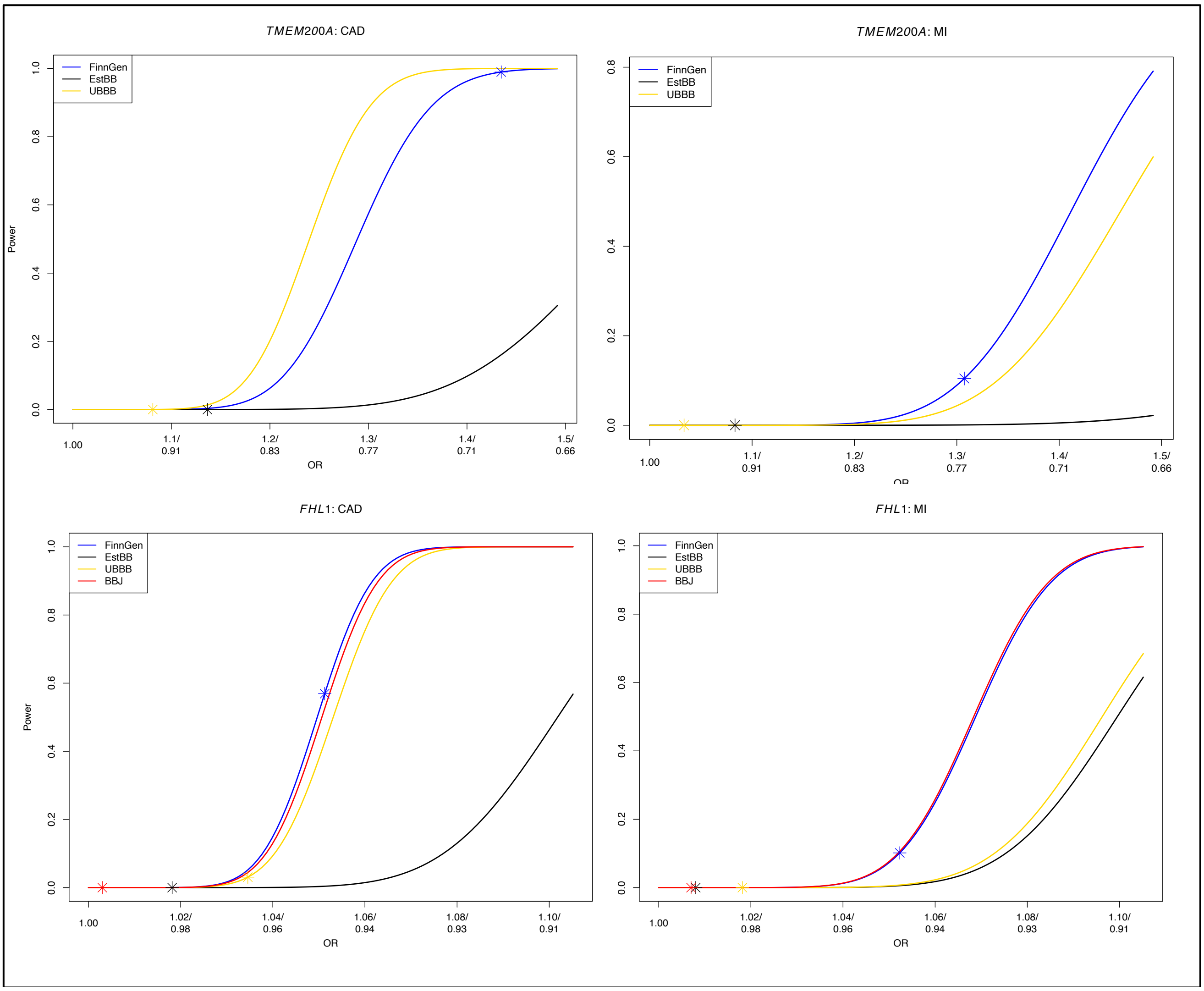

Supplementary Figure 5 (legend): *Post hoc power calculations for rs118042209 in TMEM200A and rs5974585 in FHL1. Power was calculated as a function of effect size for each cohort scenario (N cases and controls, AF). The actual effect sizes are marked as stars.*

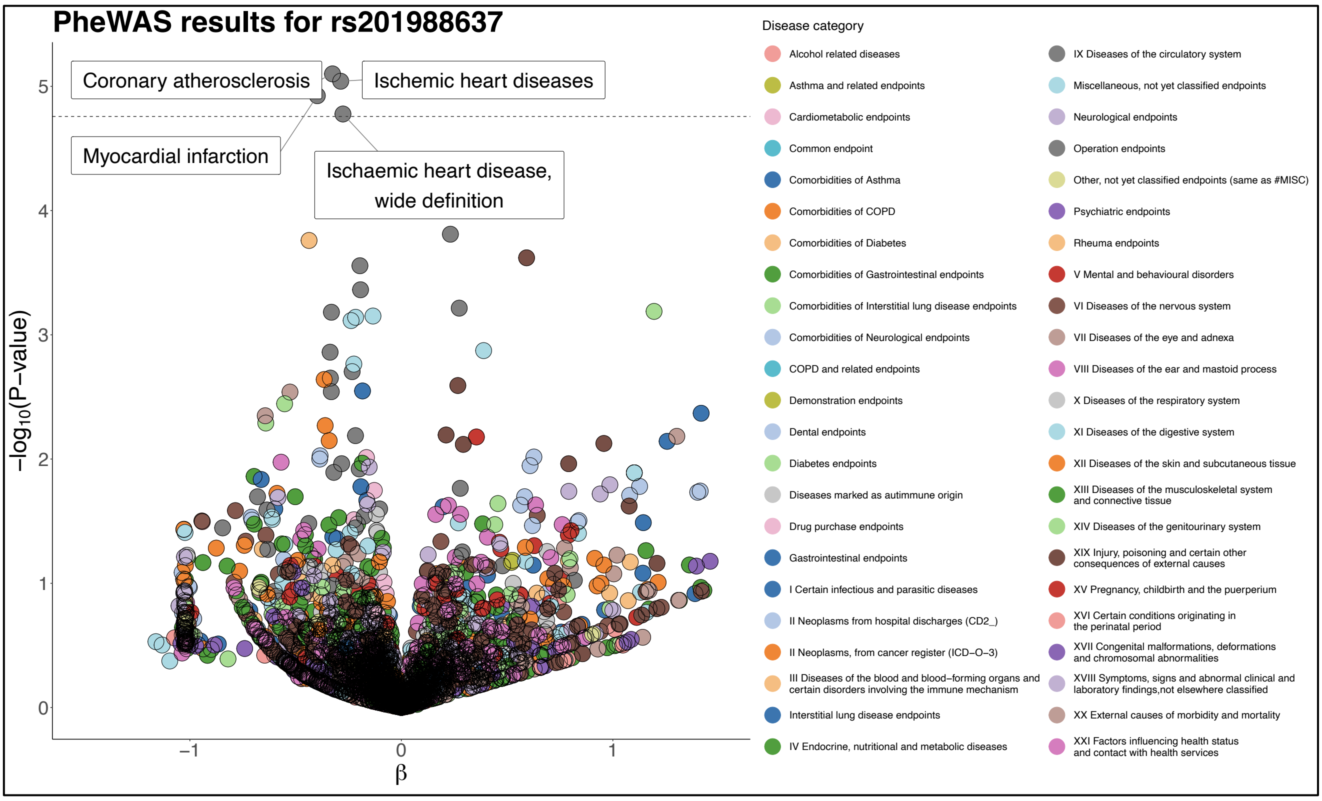

Supplementary Figure 6: *Phenome-wide association study (PheWAS) results for rs201988637. Total number of endpoints is 2 861. Dashed line represents the phenome-wide significance threshold, multiple testing corrected by the number of endpoints = 0.05/ 2 861 = 1.75×10^{-5}. All endpoints reaching that threshold are labelled.*

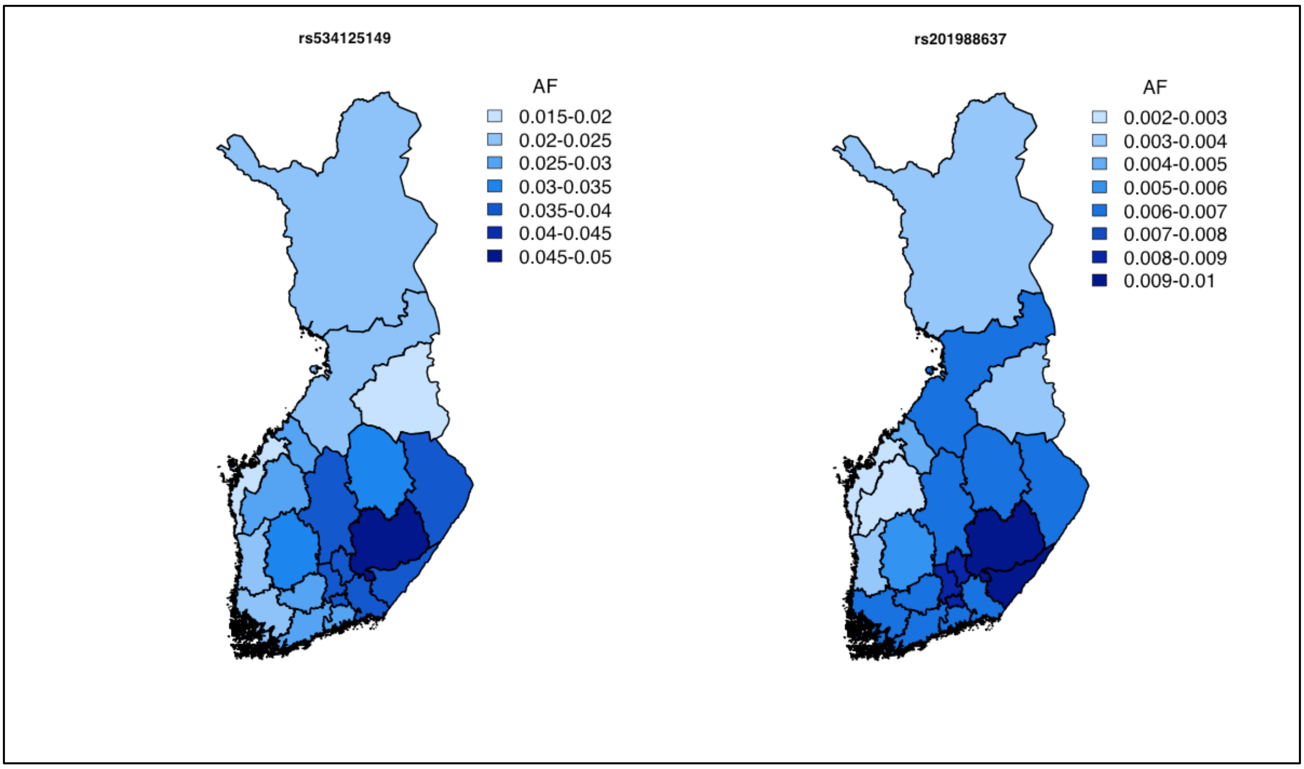

Supplementary Figure 7: *Distribution of rs534125149 (inframe insertion) and rs201988637 (splice acceptor variant) across Finland.*

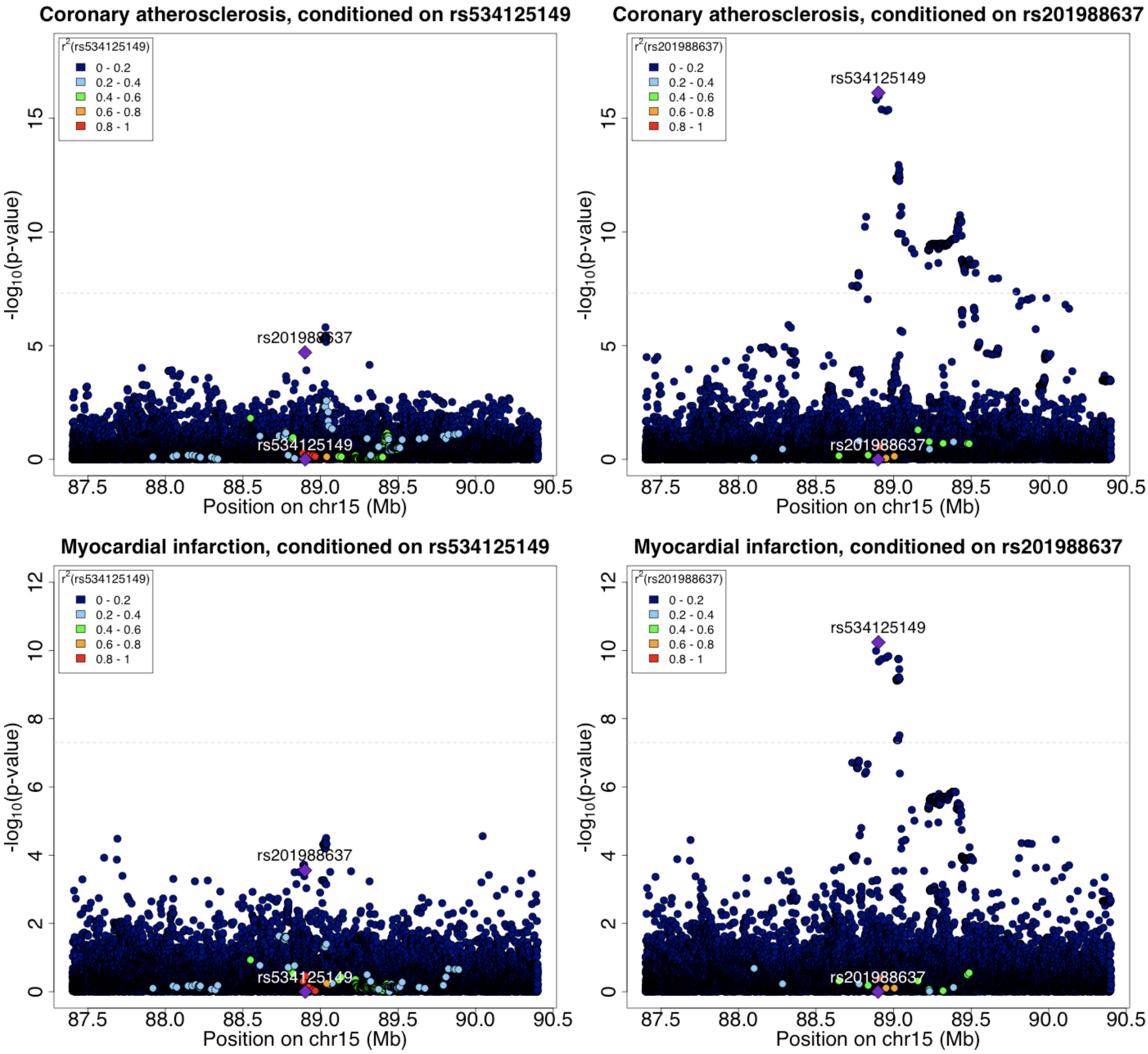

Supplementary Figure 8: *Locuszoomplots from conditional analysis on rs534125149 and rs201988637 for coronary atherosclerosis and myocardial infarction (strict definition) on ±1.5 Mb region around rs534125149.*

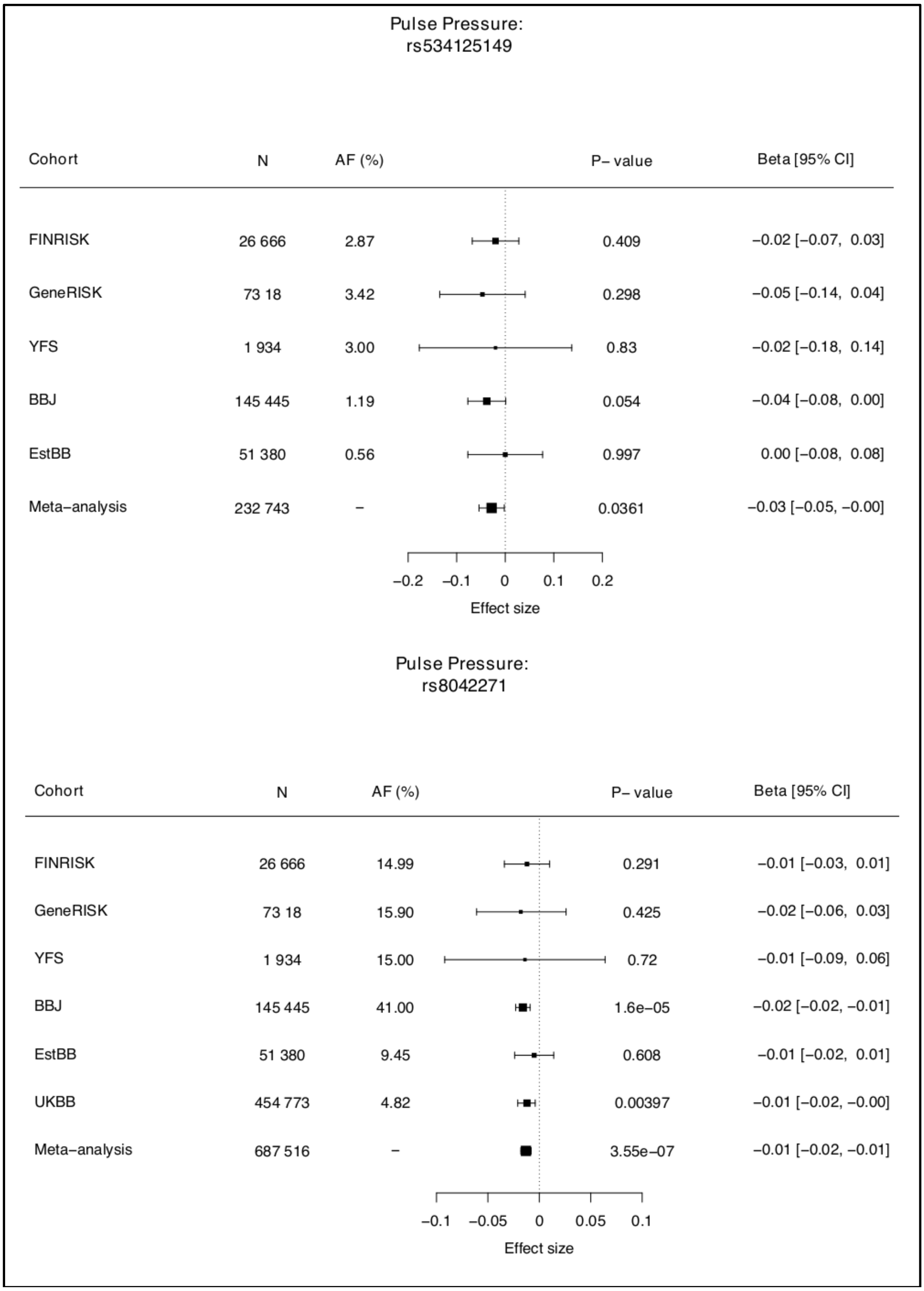

Supplementary Figure 9: *Pulse pressure association results for inframe insertion rs534125149 and common variant rs8042271 across all cohorts, where variants were available. Size of the boxes represent the sample size of the cohorts, and the lines the 95%- confidence interval. The associations were tested using linear regression and adjusting for age and sex. Pulse pressure phenotypes were inverse-rank-normalised prior analysis.*

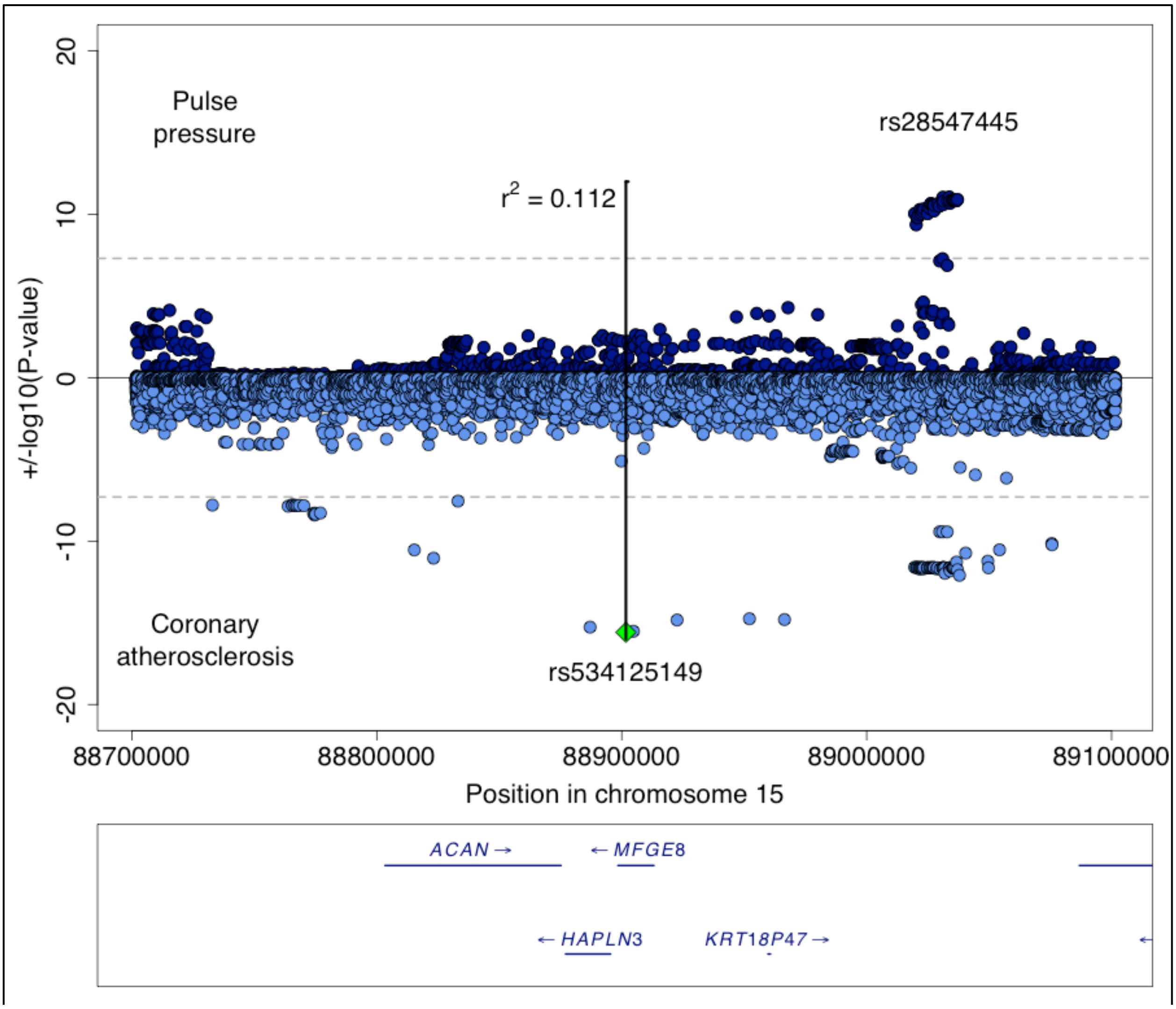

***Supplementary Figure 10**: Locuszoomplot for pulse pressure (from Evangelou et al., 2018) and coronary atherosclerosis in FinnGen in ±200 kb region around rs534125149.*

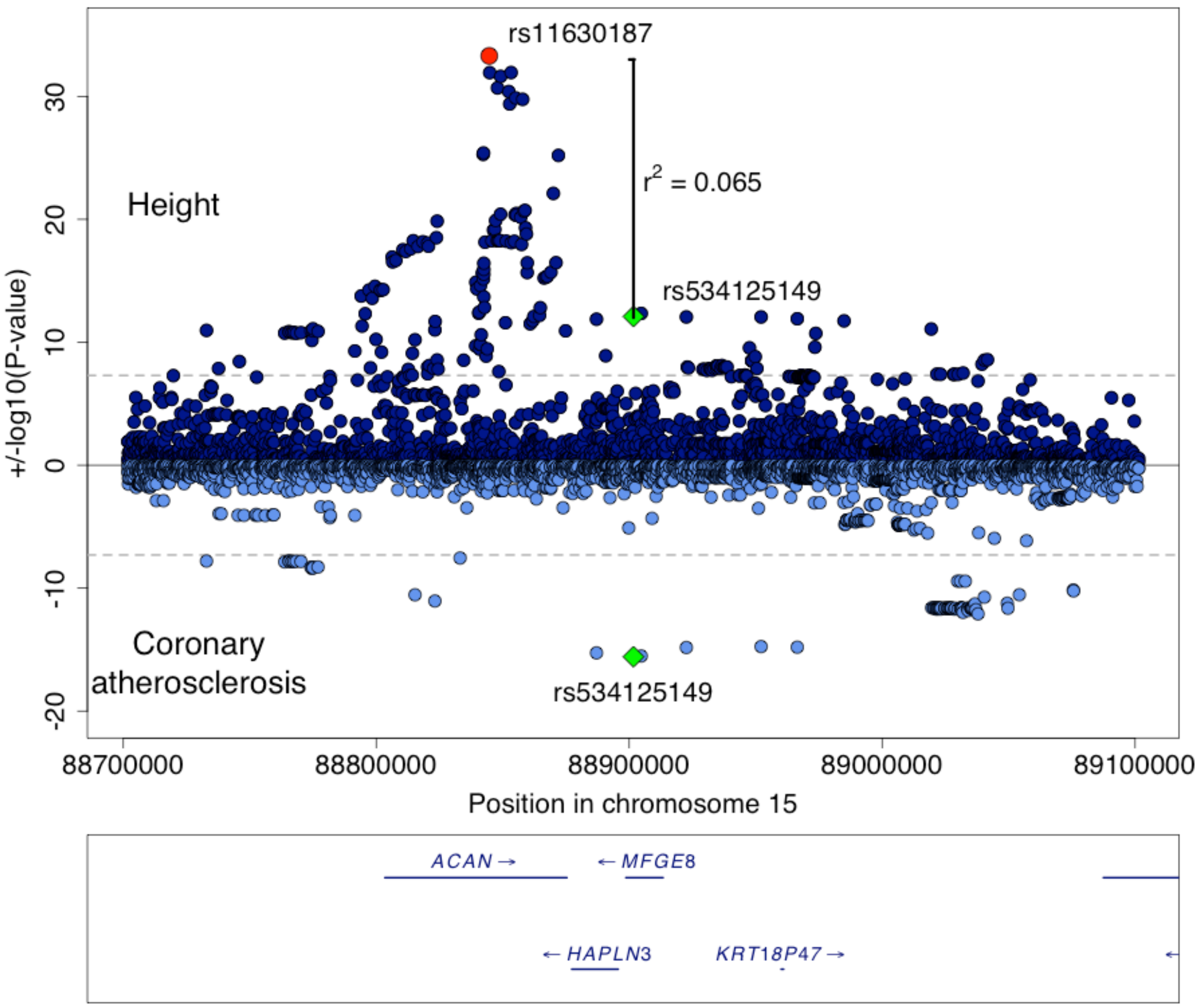

Supplementary Figure 11: *Locuszoomplot for height and coronary atherosclerosis in ±200 kb region around rs534125149.*

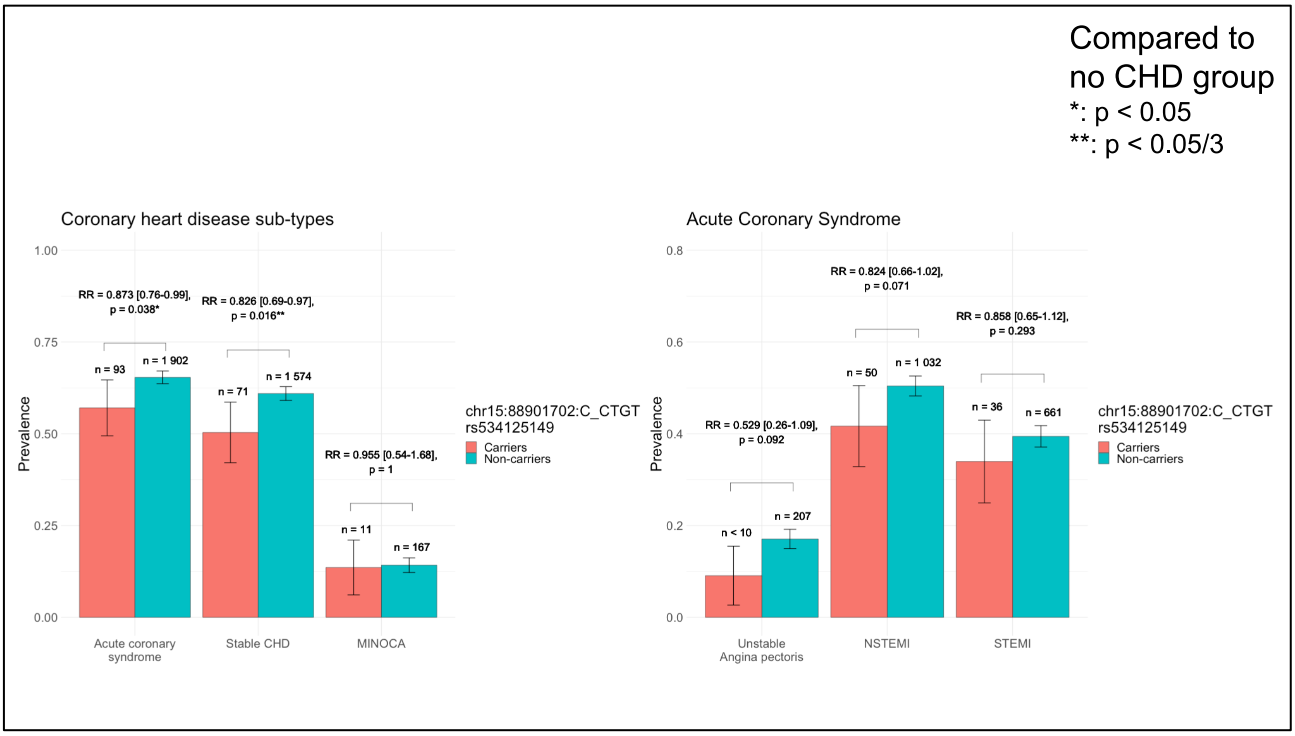

Supplementary Figure 12: *Association of rs534125149 with sub-types of coronary heart disease and acute coronary syndrome in Corogene cohort. Comparison group is always no-CHD group (n = 1 008 non-carriers and 70 carriers). The height of the bars represents the frequency of the carriers and non-carriers in each disease status group, and the error bars represents the 95 % confidence intervals. P- values are from* χ^2 *– test, calculated for each disease group against no CHD group. Source data for the figure is in Supplementary Data 1.*

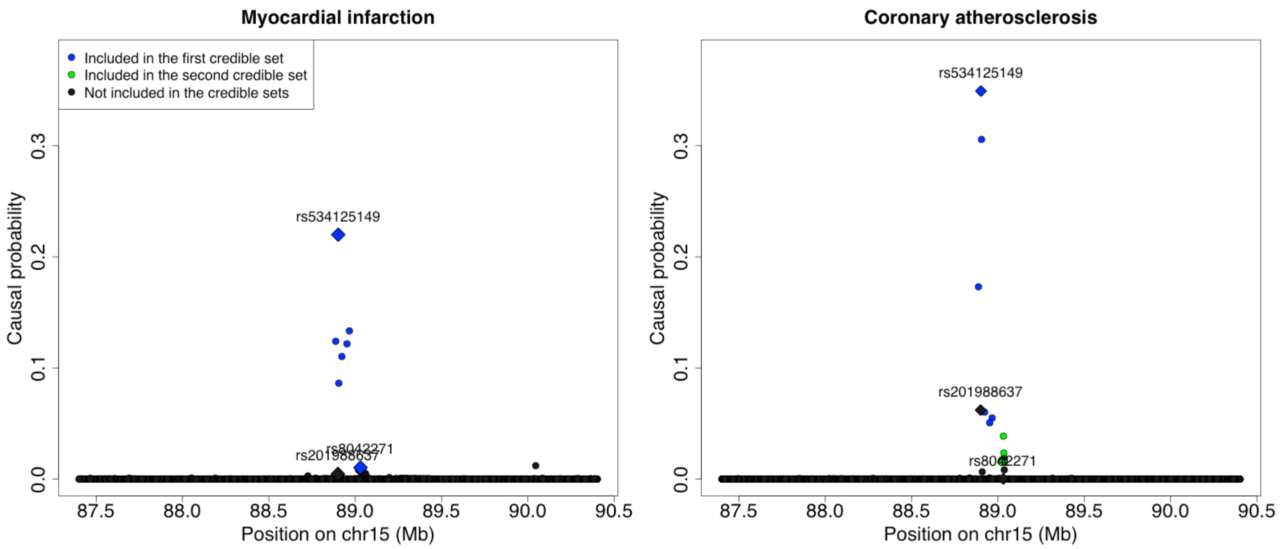

Supplementary Figure 13: *Locuszoomplots from FINEMAP analyses for myocardial infarction and coronary atherosclerosis in FinnGen R6. On the x- axis are genomic positions in build hg38, and on the y- axis the probability for being causal for each variant.*

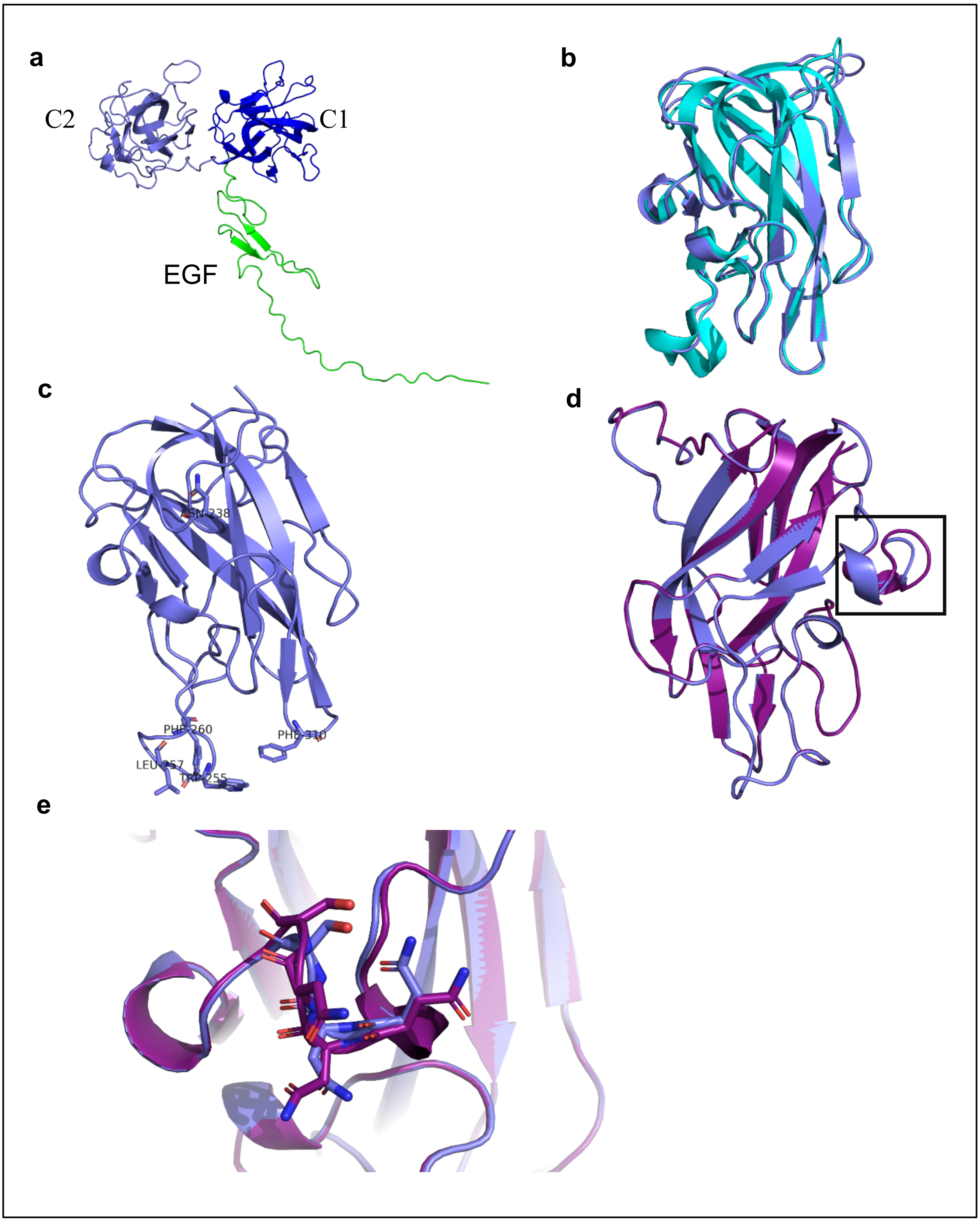

Supplementary Figure 14: *Protein modelling results for inframe insertion rs534125149. A) Predicted structure from AlphaFold of lactadherin containing EGF, C1, and C2 domains. B) Comparison of the predicted lactadherin C2 domain (blue) with the C2 domain of bovine lactadherin from x-ray crystallography (cyan, PDB ID: 2PQS). C) The loop containing the insertion variant (Asn238 reference shown) is ~20Å away from the loops participating in membrane binding. D) The predicted structure of the Asn insertion variant is shown in purple with the reference lactadherin structure shown in blue. Predicted conformational changes are localized to a single loop region. E) Comparison of the side chains in the predicted structure of the reference and Asn insertion lactadherin variant. Amino acids Asn238 to Ser240 are shown in blue. The Asn insertion variant is shown in purple*

Supplementary tables

Supplementary Table 1: *38 independent GWS associations for coronary atherosclerosis represented by the top variant. Previously unreported loci for CAD are bolded.*

SNPID (chr:pos:ref:alt)	rsid	Most severe consequence	Gene	CAD locus	Associated CAD- related trait(s)	FIN enrichment	AF	OR (P-value)
chr9:22099569:C:A	rs1537371	Intron variant	*CDKN2B-AS1*	Yes	CAD, CHD	0.8	0.42	1.25 (2.30×10^{-87})
chr6:12903725:A:G	rs9349379	Intron variant	*PHACTR1*	Yes	CAD, CHD	1.11	0.45	1.15 (9.00×10^{-33})
chr6:160589086:A:G	rs10455872	Intron variant	*LPA*	Yes	Lp(a), CAD, LDL-C	0.41	0.05	1.36 (6.70×10^{-30})
chr15:78750846:T:TAG	rs10625725	Upstream gene variant	*ADAMTS7*	Yes	CHD, DBP, CAC	0.842	0.64	0.9 (5.10×10^{-19})
chr1:55039974:G:T	rs11591147	Missense variant	*PCSK9*	Yes	LDL-C, TC, CAD, MI	3.1	0.04	0.77 (4.10×10^{-17})
chr19:11087511:C:T	rs17248720	Upstream gene variant	*LDLR*	Yes	LDL-C, TC, CAD	0.875	0.10	0.86 (2.00×10^{-16})
**chr15:88901702:C:CTGT**	**rs534125149**	**Inframe insertion**	***MFGE8***	**NO**	**-**	**inf**	**0.03**	**0.75 (2.60×10^{-16})**
chr4:147472512:C:T	rs1878406	Intergenic variant	*EDNRA*	Yes	PP, CAD	0.963	0.13	1.15 (1.00×10^{-15})
chr1:109275684:G:T	rs629301	3 prime UTR variant	*CELSR2*	Yes	LDL-C, TC, CAD	0.989	0.79	1.12 (1.60×10^{-15})
chr19:44908822:C:T	rs7412	Missense variant	*APOE*	Yes	CAD, LDL-C, TC	0.561	0.05	0.83 (4.20×10^{-14})
chr14:99649135:C:T	rs541148696	Intron variant	*HHIPL1*	Yes	CAD, SBP, MI	175	0.01	1.51 (9.80×10^{-14})
chr21:34221526:G:A	rs28451064	Intron variant	*AP000317.1/2*	Yes	CAD	1.14	0.15	1.12 (1.20×10-13)
chr11:116752497:TA:T	rs66505542, rs398017699	Intron variant	*BUD13*	Yes	TG, HDL-C, TC	0.97	0.82	0.9 (4.80×10^{-13})
chr13:110397276:T:C	rs9515203	Intron variant	*COL4A2*	Yes	CAD, LDL-C,	1.01	0.26	0.91 (2.50×10^{-12})
chr12:111569952:C:T	rs653178	Intron variant	*ATXN2*	Yes	BP, LDL-C, CAD	1.16	0.58	0.92 (3.40×10^{-12})
chr1:56506681:G:A	rs72664324	Intron variant	*PLPP3*	Yes	CAD, CHD, PP	1.18	0.11	0.88 (7.30×10^{-12})
chr2:203206233:A:G	rs72936353	Intron variant	*NBEAL1*	Yes	CAD	0.803	0.11	1.13 (3.40×10^{-11})
chr2:85532371:C:T	rs59877521	Intergenic variant	*MAT2A*	Yes	CAD	1.03	0.43	1.08 (1.30×10^{-10})
chr7:150993088:C:T	rs3918226	Intron variant	*NOS3*	Yes	CAD	0.916	0.07	1.15 (1.70×10^{-10})
chr7:106770331:G:A	rs12705390	Upstream gene variant	*PIK3CG*	Yes	TG	1.54	0.30	1.08 (3.00×10^{-10})
chr17:2264939:C:T	rs7209564	Intron variant	*SMG6*	Yes	CHD, SBP, BMI	0.973	0.72	1.08 (4.00×10^{-10})
chr1:37950638:C:T	rs72661887	Upstream gene variant	*INPP5B*	Yes	SBP, PP	1.24	0.54	1.07 (6.00×10^{-10})
chr3:138369222:G:C	rs1199337	Intron variant	*MRAS*	Yes	CAFD CHD, BMI	1	0.14	1.11 (6.80×10^{-10})
chr20:45910063:C:A	rs201557719	Missense variant	*PLTP*	Yes	HDL-C, TG	inf	0.0023	2.01 (1.40×10^{-09})
**chr6:130483492:A:G**	**rs118042209**	**Intergenic variant**	***TMEM200A***	**NO**	**-**	**0.79**	**0.01**	**0.7 (1.90×10^{-09})**
chr7:19013110:G:A	rs57301765	Intergenic variant	*TWIST1*	Yes	PP, CAD, SBP	1.3	0.19	1.09 (2.10×10^{-09})
chr12:54120216:A:T	rs11170821	Non coding transcript exon variant	*SMUG1*	Yes	CAD, BMI	2.21	0.06	1.15 (2.40×10^{-09})
chr5:108716278:T:C	rs6867978	Intergenic variant	*FER*	Yes	SBP, PP	0.854	0.29	0.93 (2.80×10^{-09})
chr12:57146968:C:G	rs7968719	Intron variant	*LRP1*	Yes	CAD, BMI	0.991	0.52	1.07 (3.50×10^{-09})
chr6:32204288:T:G	rs1044506	Synonymous variant	*NOTCH4*	Yes	BMI	1.01	0.87	1.1 (6.80×10^{-09})
chr6:133696717:G:A	rs34537042	Intron variant	*EYA4*	Yes	TG, AF, WHR	0.938	0.18	1.09 (7.50×10^{-09})
chr15:85610093:A:G	rs16942194	Intron variant	*AKAP13*	Yes	SBP, PP, CAD	2.09	0.10	0.9 (8.10×10^{-09})
chr4:41669720:A:T	rs141987800	Intron variant	*LIMCH1*	Yes	LDL-C, Statin response	0.52	0.004	1.75 (9.40×10^{-09})
chr8:22172265:C:A	rs73225841	Intron variant	*BMP1*	Yes	CAD	2.65	0.15	1.1 (1.20×10^{-08})
chr2:43845437:G:T	rs4299376	Intron variant	*ABCG8*	Yes	LDL-C, TC, CAD	1.16	0.78	0.93 (1.70×10^{-08})
**chrX:136194941:C:G**	**rs5974585**	**Intron variant**	***FHL1***	**NO**	**-**	**1.25**	**099**	**0.95 (2.55×10^{-08})**
chr8:125487789:C:G	rs28601761	Intron variant	*TRIB1*	Yes	TG, HDL-C, MI	1	0.41	0.94 (2.60×10^{-08})
**chr11:57380633:A:G**	**rs764568652**	**Intron variant**	***PRG3***	**NO**	**-**	**inf**	**0.00032**	**7.72 (4.10×10^{-08})**

Supplementary Table 2: *Significant association results from the phenome-wide scan. Association results for rs534125149 and rs201988637 for 5 endpoints with p-value < 1.75×10^{-05} (PWS) for rs534125149, and with fundamental characteristics. Total number of PWS disease endpoint for rs53412514 was 14.*

Disease endpoint	Cases / Controls	rs534125149 (inframe insertion)		rs201988637 (splice acceptor)	
		OR [95% CI]	P-value	OR [95% CI]	P-value
Coronary atherosclerosis	28 598/ 222 551	0.75 [0.71-0.81]	2.63×10^{-16}	0.72 [0.63-0.83]	7.94×10^{-06}
Coronary revascularization	14 741/ 222 551	0.69 [0.63-0.76]	1.86×10^{-15}	0.72 [0.59-0.87]	6.58×10^{-04}
Ischemic heart diseases	37 854/ 222 551	0.80 [0.75-0.84]	4.26×10^{-14}	0.75 [0.66-0.85]	9.10×10^{-06}
Major coronary heart disease event	25 707/ 234 698	0.79 [0.73-0.84]	1.12×10^{-11}	0.79 [0.68-0.92]	1.98×10^{-03}
Myocardial infarction, strict (only main diagnoses accepted)	14 305/ 222 551	0.74 [0.68-0.81]	1.95×10^{-11}	0.69 [0.58-0.83]	9.62×10^{-05}
Statin medication	86 466/ 173 939	0.90 [0.86-0.94]	1.54×10^{-05}	1.01 [0.91-1.12]	0.82

Supplementary Table 3: *Results for sensitivity analysis for survival analysis on first even of MI. Cox- proportional hazards model has been used, and all the results are for binary variable carrier/ non- carrier for either inframe insertion rs534125149 or splice acceptor variant rs534125149.*

Covariates	HR [95 % CI]	P- value
None	0.779 [0.725-0.838]	1.16×10^{-11}
Sex	0.774 [0.720-0.832]	3.30×10^{-12}
BMI	0.789 [0.728-0.854]	5.40×10^{-09}
T2D	0.771 [0.716-0.831]	7.71×10^{-12}
Statins	0.788 [0.733-0.847]	8.71×10^{-11}
Smoking	0.830 [0.749-0.919]	3.38×10^{-04}
All above	0.833 [0.742-0.935]	1.99×10^{-03}

Supplementary Table 4: *Characteristics of variants in MFGE8.*

Chr	Position[a]	Alleles (Ref/Alt)	rsid	Af (alt)	Info	Most severe consequence	ld with rs534125149	ld with rs201988637	ld with rs8042271
15	88901702	C/CTGT	rs534125149	0.029	0.990	Inframe insertion	1	0.0001	0.1536
15	88899813	T/G	rs201988637	0.006	0.986	Splice acceptor variant	0.0001	1	0.0010
15	89030987	G/A	rs8042271	0.148	0.988	Intergenic variant	0.1536	0.0010	1

[a] Positions are in build 38

Supplementary Table 5: *95% credible set for myocardial infarction (strict definition) from FINEMAP. Inframe insertion (rs534125149) and previously known common variant (rs8042271) are bolded.*

SNPID (chr:pos:ref:alt)	rsid	Causal probability	ld with rs534125149
**chr15:88901702:C:CTGT**	**rs534125149**	**0.250**	**1.000**
chr15:88966448:A:C	rs12594129	0.156	0.962
chr15:88952146:C:T	rs150599388	0.141	0.963
chr15:88887075:G:T	rs565898017	0.136	0.991
chr15:88922596:C:T	rs117951873	0.125	0.967
chr15:88904754:C:T	rs191156695	0.089	0.981
**chr15:89030987:G:A**	**rs8042271**	**0.003**	**0.154**
chr15:89032865:T:A	rs56015348	0.003	0.154
chr15:89029825:A:C	rs2003967	0.003	0.154
chr15:89037068:C:G	rs2351254	0.002	0.112
chr15:89034032:T:C	rs8035408	0.002	0.112
chr15:89036690:G:C	rs7164299	0.002	0.114
chr15:89026237:T:G	rs4932463	0.002	0.112
chr15:89026272:G:C	rs4932208	0.002	0.112
chr15:89028938:C:T	rs1550476	0.002	0.112
chr15:89022026:A:C	rs1807214	0.002	0.112
chr15:89023347:T:C	rs730657	0.002	0.112
chr15:89024863:C:A	rs34166180	0.002	0.112
chr15:89030984:A:G	rs8023801	0.002	0.112
chr15:89031951:T:C	rs7162318	0.002	0.112
chr15:89033730:T:C	rs28547445	0.002	0.112
chr15:89021271:A:T	rs12592098	0.002	0.112
chr15:89022834:C:T	rs735836	0.002	0.112
chr15:89022640:T:A	rs735837	0.002	0.112
chr15:89024874:A:AAAG	rs10683082	0.002	0.112
chr15:89024799:T:G	rs9672658	0.002	0.112
chr15:89026138:A:C	rs4932207	0.002	0.112
chr15:89027167:G:C	rs28737395	0.002	0.112
chr15:89026859:A:C	rs8041298	0.002	0.112
chr15:89027456:A:G	rs12437794	0.002	0.112
chr15:89027517:T:C	rs7169670	0.002	0.112
chr15:89027435:G:C	rs7162610	0.002	0.112

Supplementary Table 6*: 95% credible sets for coronary atherosclerosis. Inframe insertion variant (rs534125149) is bolded. The LD's with the lead variant (variant with highest probability of being causal) in each credible set are reported.*

Credible set 1				Credible set 2			
SNPID chr:pos:ref:alt	rsid	Causal prob	ld with rs534125149	SNPID chr:pos:ref:alt	rsid	Causal prob	ld with rs7162326
**chr15:88901702:C:CTGT**	**rs534125149**	**0.318**	**1.000**	chr15:89031969:T:C	rs7162326	0.048	1.000
chr15:88904754:C:T	rs191156695	0.309	0.981	chr15:89034032:T:C	rs8035408	0.028	0.952
chr15:88887075:G:T	rs565898017	0.184	0.991	chr15:89037068:C:G	rs2351254	0.025	0.950
chr15:88922596:C:T	rs117951873	0.064	0.967	chr15:89033989:G:A	rs8029102	0.021	0.952
chr15:88966448:A:C	rs12594129	0.059	0.962	chr15:89030984:A:G	rs8023801	0.021	0.952
chr15:88952146:C:T	rs150599388	0.055	0.963	chr15:89031951:T:C	rs7162318	0.021	0.952
				chr15:89035227:A:T	rs8037109	0.021	0.952
				chr15:89033730:T:C	rs28547445	0.021	0.952
				chr15:89031195:C:G	rs2083459	0.021	0.952
				chr15:89031187:T:C	rs2083458	0.021	0.952
				chr15:89026237:T:G	rs4932463	0.021	0.952
				chr15:89026272:G:C	rs4932208	0.021	0.952
				chr15:89028938:C:T	rs1550476	0.021	0.952
				chr15:89025698:A:G	rs4932206	0.021	0.952
				chr15:89035651:A:G	rs7182265	0.021	0.952
				chr15:89035631:A:T	rs7182131	0.021	0.952
				chr15:89031158:A:G	rs2083457	0.021	0.952
				chr15:89031253:T:C	rs2083460	0.021	0.952
				chr15:89031209:GT:G	rs35590540	0.021	0.952
				chr15:89031714:T:TG	rs35681620/ rs397714366	0.021	0.952
				chr15:89026138:A:C	rs4932207	0.021	0.952
				chr15:89035106:T:C	rs8041247	0.021	0.952
				chr15:89035116:G:C	rs8035119	0.021	0.952
				chr15:89035492:T:C	rs6496549	0.021	0.952
				chr15:89024863:C:A	rs34166180	0.021	0.952
				chr15:89036232:A:G	rs12593383	0.021	0.952
				chr15:89035688:A:T	rs7182288	0.021	0.952
				chr15:89024874:A:AAAG	rs10683082	0.021	0.952
				chr15:89024799:T:G	rs9672658	0.021	0.952
				chr15:89027167:G:C	rs28737395	0.021	0.952
				chr15:89026859:A:C	rs8041298	0.021	0.952
				chr15:89027456:A:G	rs12437794	0.021	0.952
				chr15:89027435:G:C	rs7162610	0.021	0.952
				chr15:89027517:T:C	rs7169670	0.021	0.952
				chr15:89021727:T:C	rs734780	0.021	0.952
				chr15:89022026:A:C	rs1807214	0.020	0.952
				chr15:89021271:A:T	rs12592098	0.020	0.952
				chr15:89022834:C:T	rs735836	0.020	0.952
				chr15:89022640:T:A	rs735837	0.020	0.952
				chr15:89023347:T:C	rs730657	0.020	0.952
				chr15:89022255:C:T	rs8037001	0.020	0.952
				chr15:89020702:T:C	rs28731436	0.020	0.952
				chr15:89020713:A:G	rs28735097	0.020	0.952
				chr15:89020231:C:G	rs1985691	0.020	0.952
				chr15:89019583:C:G	rs28687065	0.020	0.951

Supplementary Table 7: *Association results for inframe insertion rs534125149, splice acceptor variant rs201988637 and common variants rs8042271 and rs734780 in MFGE8 discussed for coronary atherosclerosis and MI, both separately and all three variants in the same model. Logistic regression adjusted for age, sex, genotyping batch and PC1-10 have been applied.*

Endpoint	rsid	SNPID (chr:position:ref:alt)	Separately		All in same model	
			OR [CI]	P	OR [CI]	P
rs8042271						
Coronary atherosclerosis	rs534125149	chr15:88901702:C:CTGT	0.76 [0.71-0.81]	8.30×10^{-17}	0.79 [0.74-0.85]	1.14×10^{-10}
	rs201988637	chr15:88899813:T:G	0.76 [0.66-0.87]	1.04×10^{-4}	0.75 [0.65-0.86]	4.41×10^{-5}
	rs8042271	chr15:89030987:G:A	0.91 [0.88-0.94]	1.49×10^{-10}	0.94 [0.91-0.97]	4.56×10^{-4}
Myocardial Infarction, strict	rs534125149	chr15:88901702:C:CTGT	0.74 [0.68-0.81]	6.17×10^{-11}	0.79 [0.72-0.88]	5.42×10^{-6}
	rs201988637	chr15:88899813:T:G	0.72 [0.59-0.87]	6.66×10^{-4}	0.70 [0.57-0.85]	2.87×10^{-4}
	rs8042271	chr15:89030987:G:A	0.88 [0.84-0.91]	1.82×10^{-10}	0.91 [0.87-0.95]	2.30×10^{-5}
rs734780						
Coronary atherosclerosis	rs534125149	chr15:88901702:C:CTGT	0.76 [0.71-0.81]	8.30×10^{-17}	0.80 [0.75-0.86]	2.83×10^{-10}
	rs201988637	chr15:88899813:T:G	0.76 [0.66-0.87]	1.04×10^{-4}	0.73 [0.64-0.84]	1.13×10^{-5}
	rs734780	chr15:89021727:T:C	0.90 [0.88-0.93]	2.36×10^{-12}	0.93 [0.91-0.96]	1.62×10^{-6}
Myocardial Infarction, strict	rs534125149	chr15:88901702:C:CTGT	0.74 [0.68-0.81]	6.17×10^{-11}	0.78 [0.71-0.86]	3.70×10^{-7}
	rs201988637	chr15:88899813:T:G	0.72 [0.59-0.87]	6.66×10^{-4}	0.67 [0.58-0.83]	1.04×10^{-4}
	rs734780	chr15:89021727:T:C	0.89 [0.86-0.92]	9.19×10^{-10}	0.92 [0.88-0.95]	1.31×10^{-5}
