# Supplementary material for: Inframe insertion and splice site variants in MFGE8 associate with protection against coronary atherosclerosis
Source: Commun Biol. 2022 Aug 17;5:802. doi: 10.1038/s42003-022-03552-0 (PMC9385630; doi:10.1038/s42003-022-03552-0)
Supplement: Supplementary file 3 — Reporting Summary [file 42003_2022_3552_MOESM3_ESM.pdf]

nature research

Corresponding author(s): Samuli Ripatti

Last updated by author(s): 31.05.2022

Reporting Summary

Nature Research wishes to improve the reproducibility of the work that we publish. This form provides structure for consistency and transparency in reporting. For further information on Nature Research policies, see our Editorial Policies and the Editorial Policy Checklist.

Statistics

For all statistical analyses, confirm that the following items are present in the figure legend, table legend, main text, or Methods section.

n/a	Confirmed	
☐	☒	The exact sample size (*n*) for each experimental group/condition, given as a discrete number and unit of measurement
☐	☒	A statement on whether measurements were taken from distinct samples or whether the same sample was measured repeatedly
☐	☒	The statistical test(s) used AND whether they are one- or two-sided<br>*Only common tests should be described solely by name; describe more complex techniques in the Methods section.*
☐	☒	A description of all covariates tested
☐	☒	A description of any assumptions or corrections, such as tests of normality and adjustment for multiple comparisons
☐	☒	A full description of the statistical parameters including central tendency (e.g. means) or other basic estimates (e.g. regression coefficient) AND variation (e.g. standard deviation) or associated estimates of uncertainty (e.g. confidence intervals)
☐	☒	For null hypothesis testing, the test statistic (e.g. *F*, *t*, *r*) with confidence intervals, effect sizes, degrees of freedom and *P* value noted<br>*Give P values as exact values whenever suitable.*
☒	☐	For Bayesian analysis, information on the choice of priors and Markov chain Monte Carlo settings
☐	☒	For hierarchical and complex designs, identification of the appropriate level for tests and full reporting of outcomes
☒	☐	Estimates of effect sizes (e.g. Cohen's *d*, Pearson's *r*), indicating how they were calculated

Our web collection on statistics for biologists contains articles on many of the points above.

Software and code

Policy information about availability of computer code

Data collection	No software was used.
Data analysis	All disease endpoints were analyzed using Scalable and Accurate Implementation of Generalized mixed model, SAIGE (version 0.39.1). aOther statistical analyses have been performed using R, version 3.6.3.

For manuscripts utilizing custom algorithms or software that are central to the research but not yet described in published literature, software must be made available to editors and reviewers. We strongly encourage code deposition in a community repository (e.g. GitHub). See the Nature Research guidelines for submitting code & software for further information.

Data

Policy information about availability of data

All manuscripts must include a data availability statement. This statement should provide the following information, where applicable:

- Accession codes, unique identifiers, or web links for publicly available datasets
- A list of figures that have associated raw data
- A description of any restrictions on data availability

The FinnGen genotype and phenotype data may be accessed through Finnish Biobanks' FinnBB portal (www.finbb.fi) and THL Biobank data through THL Biobank (https://thl.fi/en/web/thl-biobank).

The BBJ summary statistics are available at the National Bioscience Database Center (NBDC) Human Database (accession code: hum0197) and at the GWAS catalog (https://www.ebi.ac.uk/gwas/home). They are also browseable at our PheWeb website (https://pheweb.jp/). The variant rs534125149 was originally excluded from the publicly available GWAS summary statistics. Its associations were reported in Supplementary Figure 4. The BBJ genotype data is accessible on request at the Japanese Genotype-phenotype Archive (http://trace.ddbj.nig.ac.jp/jga/index_e.html) with accession code JGAD00000000123 and JGAS00000000114.

Genotype and phenotype data are available from the Estonian Biobank (https://genomics.ut.ee/en/biobank.ee/data-access) upon request.

The dataset supporting the conclusions of this article were obtained from the Cardiovascular Risk in Young Finns Study which comprises health related participant data. The use of data is restricted under the regulations on professional secrecy (Act on the Openness of Government Activities, 612/1999) and on sensitive personal data (Personal Data Act, 523/1999, implementing the EU data protection directive 95/46/EC). Due to these restrictions, the data cannot be stored in public repositories or otherwise made publicly available. Data access may be permitted on a case-by-case basis upon request only. Data sharing outside the group is done in collaboration with YFS group and requires a data-sharing agreement. Investigators can submit an expression of interest to the chairman of the publication committee Professor Mika Kähönen (Tampere University, Finland) or Professor Terho Lehtimäki (Tampere University, Finland).
The FinnGen genotype and phenotype data may be accessed through Finnish Biobanks' FinnBB portal (www.finbb.fi) and THL Biobank data through THL Biobank (https://thl.fi/en/web/thl-biobank).

The BBJ summary statistics are available at the National Bioscience Database Center (NBDC) Human Database (accession code: hum0197) and at the GWAS catalog (https://www.ebi.ac.uk/gwas/home). They are also browseable at our PheWeb website (https://pheweb.jp/). The variant rs534125149 was originally excluded from the publicly available GWAS summary statistics. Its associations were reported in Supplementary Figure 4. The BBJ genotype data is accessible on request at the Japanese Genotype-phenotype Archive (http://trace.ddbj.nig.ac.jp/jga/index_e.html) with accession code JGAD00000000123 and JGAS00000000114.

Genotype and phenotype data are available from the Estonian Biobank (https://genomics.ut.ee/en/biobank.ee/data-access) upon request.

The dataset supporting the conclusions of this article were obtained from the Cardiovascular Risk in Young Finns Study which comprises health related participant data. The use of data is restricted under the regulations on professional secrecy (Act on the Openness of Government Activities, 612/1999) and on sensitive personal data (Personal Data Act, 523/1999, implementing the EU data protection directive 95/46/EC). Due to these restrictions, the data cannot be stored in public repositories or otherwise made publicly available. Data access may be permitted on a case-by-case basis upon request only. Data sharing outside the group is done in collaboration with YFS group and requires a data-sharing agreement. Investigators can submit an expression of interest to the chairman of the publication committee Professor Mika Kähönen (Tampere University, Finland) or Professor Terho Lehtimäki (Tampere University, Finland).

Field-specific reporting

Please select the one below that is the best fit for your research. If you are not sure, read the appropriate sections before making your selection.

☒ Life sciences ☐ Behavioural & social sciences ☐ Ecological, evolutionary & environmental sciences

For a reference copy of the document with all sections, see nature.com/documents/nr-reporting-summary-flat.pdf

Life sciences study design

All studies must disclose on these points even when the disclosure is negative.

Sample size	260 405 for FinnGen R6. Data recruiting from biobanks is ongoing, planning to recruit 500 000 samples. This is the number of the most current data release of FinnGen at time of the analysis performed.
Data exclusions	No data were excluded from the analysis.
Replication	Association results were replicated in BioBank Japan, Estonian Biobank, Young Finns Study and UK Biobank once. All associtions of MFGE8 variants were successfully replicated.
Randomization	Participants were allocated into case and controls groups of diseases based on diagnosis yielded from health and death registries. All statistical models were adjusted for age, sex, genotyping batch and first ten principal component.
Blinding	This study based completely on cohort samples. All allocations on disease groups have been performed after recruitment from electronic health registries, and therefore all investigators have been blinded to them.

Reporting for specific materials, systems and methods

We require information from authors about some types of materials, experimental systems and methods used in many studies. Here, indicate whether each material, system or method listed is relevant to your study. If you are not sure if a list item applies to your research, read the appropriate section before selecting a response.

Materials & experimental systems

n/a	Involved in the study
☒	☐ Antibodies
☒	☐ Eukaryotic cell lines
☒	☐ Palaeontology and archaeology
☒	☐ Animals and other organisms
☐	☒ Human research participants
☒	☐ Clinical data
☒	☐ Dual use research of concern

Methods

n/a	Involved in the study
☒	☐ ChIP-seq
☒	☐ Flow cytometry
☒	☐ MRI-based neuroimaging

Human research participants

Policy information about studies involving human research participants

Population characteristics	FinnGen includes prospective epidemiological and disease-based cohorts as well as hospital biobank samples. FinnGen covers whole Finland geographically and all age groups, with unbiased sex distribution.
Recruitment	FinnGen includes prospective epidemiological and disease-based cohorts as well as hospital biobank samples.
Ethics oversight	Patients and control subjects in FinnGen provided informed consent for biobank research, based on the Finnish Biobank Act. Alternatively, separate research cohorts, collected prior the Finnish Biobank Act came into effect (in September 2013) and start of FinnGen (August 2017), were collected based on study-specific consents and later transferred to the Finnish biobanks after approval by Fimea, the National Supervisory Authority for Welfare and Health. Recruitment protocols followed the biobank protocols approved by Fimea. The Coordinating Ethics Committee of the Hospital District of Helsinki and Uusimaa (HUS) approved the FinnGen study protocol Nr HUS/990/2017.<br><br>The FinnGen study is approved by Finnish Institute for Health and Welfare, Digital and population data service agency, the Social Insurance Institution and Statistics Finland. Permit numbers can be found in the Ethics statement section in the manuscript.

Note that full information on the approval of the study protocol must also be provided in the manuscript.
